# Spectral normative modeling of brain structure

**DOI:** 10.1101/2025.01.16.25320639

**Published:** 2025-01-21

**Authors:** Sina Mansour L, Maria A Di Biase, Hongwei Yan, Aihuiping Xue, Narayanaswamy Venketasubramanian, Eddie Chong, Aaron Alexander-Bloch, Christopher Chen, Juan Helen Zhou, B.T. Thomas Yeo, Andrew Zalesky

**Affiliations:** 1 Centre for Sleep & Cognition & Centre for Translational Magnetic Resonance Research, Yong Loo Lin School of Medicine, National University of Singapore, Singapore; 2 Systems Neuroscience Lab, Department of Psychiatry, The University of Melbourne, Parkville, Victoria, Australia; 3 Stem Cell Disease Modelling Lab, Department of Anatomy and Physiology, The University of Melbourne, Parkville, Victoria, Australia; 4 Psychiatry Neuroimaging Laboratory, Department of Psychiatry, Brigham and Women’s Hospital, Harvard Medical School, Boston, USA; 5 Raffles Neuroscience Centre, Raffles Hospital, Singapore; 6 Memory Aging and Cognition Centre, National University Health System, Singapore, Singapore; 7 Brain-Gene Development Laboratory, Lifespan Brain Institute at Children’s Hospital of Philadelphia and University of Pennsylvania, Philadelphia, PA, United States; 8 Department of Child and Adolescent Psychiatry and Behavioral Science, Children’s Hospital of Philadelphia, Philadelphia, PA United States; 9 Department of Psychiatry, University of Pennsylvania, Philadelphia, PA United States; 10 Department of Pharmacology, Yong Loo Lin School of Medicine, National University of Singapore, Singapore; 11 Department of Electrical and Computer Engineering, National University of Singapore, Singapore; 12 Integrative Sciences and Engineering Programme (ISEP), National University of Singapore, Singapore; 13 Department of Medicine, Healthy Longevity Translational Research Programme, Human Potential Translational Research Programme & Institute for Digital Medicine (WisDM), Yong Loo Lin School of Medicine, National University of Singapore, Singapore; 14 N.1 Institute for Health, National University of Singapore, Singapore; 15 Martinos Center for Biomedical Imaging, Massachusetts General Hospital, Charlestown, MA, United States; 16 Department of Biomedical Engineering, The University of Melbourne, Parkville, Victoria, Australia

**Keywords:** Normative Modeling, Brain Charts, Graph Signal Processing, Brain Eigenmodes

## Abstract

Normative modeling in neuroscience aims to characterize interindividual variation in brain phenotypes and thus establish reference ranges, or brain charts, against which individual brains can be compared. Normative models are typically limited to coarse spatial scales due to computational constraints, limiting their spatial specificity. They additionally depend on fixed regions from fixed parcellation atlases, restricting their adaptability to alternative parcellation schemes. To overcome these key limitations, we propose *spectral normative modeling* (SNM), which leverages brain eigenmodes for efficient spatial reconstruction to generate normative ranges for arbitrary new regions of interest. Benchmarking against conventional counterparts, SNM achieves a 98.3% speedup in computing accurate normative ranges across spatial scales, from millimeters to the whole brain. We demonstrate its utility by elucidating high-resolution individual cortical atrophy patterns and characterizing the heterogeneous nature of neurodegeneration in Alzheimer’s disease. SNM lays the groundwork for a new generation of spatially precise brain charts, offering substantial potential to drive advances in individualized precision medicine.

## 1 Introduction

Normative modeling aims to estimate reference ranges of normative population-wide variation in a phenotype of interest^1–3^. Individuals can be benchmarked to an established normative range for their age and sex to determine whether they fall outside a critical healthy range^4,5^. Normative modeling is a major goal in neuroscience and such models have been established for numerous whole-brain and regional phenotypes, such as cortical thickness or volume^6^. Prior research has demonstrated how such methods can accurately model the heterogeneous nature of these deviation patterns in brain structure^4,6^. Consequently, normative techniques hold significant promise in advancing precision medicine^7–14^.

Normative brain charts for magnetic resonance imaging (MRI) phenotypes can in principle be established at the highly detailed spatial resolution at which the MRI scan is acquired. This would enable highly localized and spatially specific inference about an individual’s deviations in a cortical phenotype. However, thus far, concerns about computational feasibility and quality control of higher-resolution phenotypes have hindered the development of efficient normative charts with high spatial precision. Conventional normative approaches are designed to estimate ranges for a single phenotypic summary statistic, such as mean cortical thickness averaged across the whole cortex^2,15,16^. To reach higher spatial specificity, normative brain charting studies typically repeat model-fitting for regional summary statistics defined over a predetermined brain atlas^6,17^.

Alternatively, a computationally burdensome exhaustive repetition of conventional techniques over all voxels/vertices has been used to produce norms at high spatial resolution^8,13,18^. These approaches are inherently sensitive to noise at the level of single voxels, and their computational intractability becomes even more pronounced when population-wide normative models are trained using data collated from several large-scale imaging biobanks. Moreover, with current normative modeling approaches, determining a reference range for a new spatial region of interest requires fitting a separate normative model anew, a process often hindered by limited access to the original training data that is typically unavailable to end-users. Establishing a methodological framework that efficiently alleviates such limitations will enable a more principled and efficient mapping of brain charts at high spatial resolution compared to the current brute force approach.

Developing high-resolution normative models is challenging, particularly due to the high dimensionality of the feature space, i.e. hundreds of thousands of vertices on the cortical surface mesh. Moreover, spatial dependencies across vertices/voxels undermine independence assumptions and further complicate the development of models that accurately explain high-resolution statistical interdependencies^19–21^. As such, finding an appropriate low-dimensional encoding of high-resolution cortical information may enable the development of computationally tractable techniques for high-resolution normative models.

Through recent advances in brain signal processing, eigenmodes constructed from the brain’s geometry and connectivity have yielded promising basis functions that can summarize phenotypic variations on the cortical surface^22–24^. As such, eigenmodes can provide a solution to high-resolution normative modeling of brain phenotypes. Spatial variation in a cortical phenotype can be captured using a lower dimensional graph spectral embedding^25,26^. We exploit this parsimonious eigenmode basis to establish normative models on the coefficients of cortical phenotypes expressed in this lower dimensional latent space. By formulating a method that relates the normative range of an arbitrary region of interest to eigenmode normative ranges, we develop a computationally efficient method that simultaneously estimates normative ranges over multiple spatial granularities.

In this work, we introduce spectral normative modeling (SNM) as a novel method to establish normative reference brain charts that are independent of any spatial resolution or parcellation atlas. We explain how high-dimensional brain phenotypes (e.g., cortical thickness) can be summarized by a concise representation based on brain eigenmodes and detail the mathematical framework to reconstruct reference normative brain models at arbitrary spatial scales. We evaluate the performance of the proposed model relative to existing approaches to demonstrate its success in providing efficient and accurate multi-scale individual-level insights that advance the frontiers of precision normative assessments. Finally, we demonstrate the practical utility of SNM in characterizing individual deviations in cortical thickness linked to cognitive impairments in Alzheimer’s disease (AD). This emphasizes the success of SNM in generalizing to unseen samples and elucidating individual heterogeneity in cortical atrophy.

## 2 Results

### 2.1 SNM: An Efficient Framework for Multi-Scale Normative Modeling

The fundamental characteristics of many engineered and natural systems can be modeled by their structural eigenmodes, which offer a simplified yet powerful means of capturing the system’s behavior. Recent advances in neuroscience have similarly demonstrated that brain eigenmodes provide a parsimonious basis for characterizing cortical information^22,25,27^. By leveraging such basis sets, we aim to enhance both the efficiency and spatial versatility of conventional normative models. In particular, we use brain connectivity eigenmodes, which naturally extend across both cortical hemispheres while preserving key neuroanatomical landmarks, such as homotopic symmetries and the separation of cortical lobes. This is especially advantageous given that pathological brain alterations often propagate along the brain’s structural network^28–34^, making connectivity eigenmodes well suited to capture normative deviations induced by mechanisms of axonal propagation. However, it is important to note that the methodological advances introduced in this work are not limited to connectivity eigenmodes and can be readily generalized to any orthonormal basis set for information reconstruction.

**Figure. 1.**
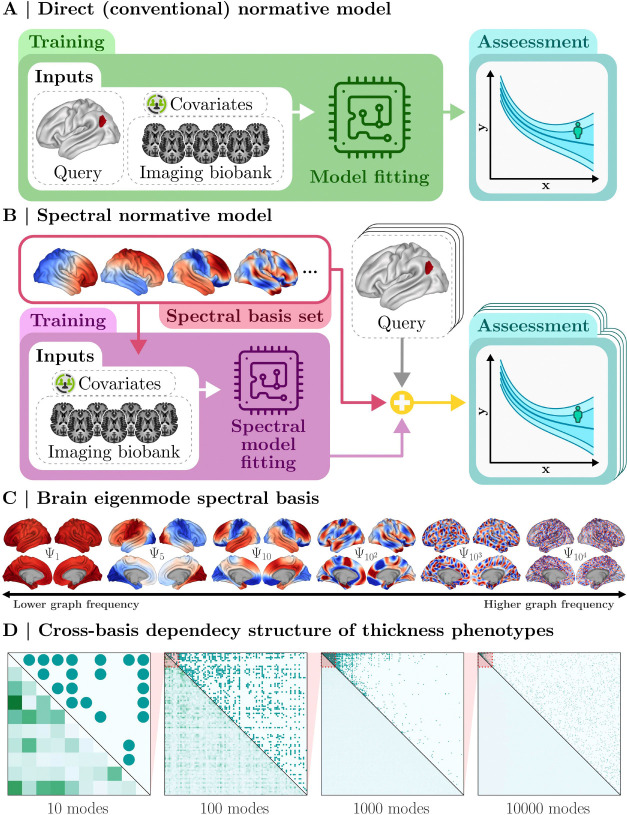
Schematic Comparison of Spectral Normative Modeling (SNM) with Conventional Normative Models. **(A)** Design diagram for conventional (direct) normative models, which require a predetermined spatial query for model training. **(B)** Design diagram of SNM, which alleviates the need for predefined spatial queries. Instead, SNM enables assessments of multiple, arbitrary, a posteriori-defined spatial queries, allowing for greater flexibility. **(C)** Cortical projections of brain connectivity eigenmodes used in SNM. Eigenmodes across a range of graph frequencies are shown, illustrating how higher frequencies capture increasingly finer spatial details. **(D)** Cross-basis correlation magnitudes of cortical thickness phenotypes encoded onto the eigenmode basis set. The sparsity of cross-mode dependencies is demonstrated through nested heatmaps, with the lower triangles representing correlation magnitudes and the upper triangles indicating suprathreshold eigenmode pairs at a correlation threshold of ρ=0.25.

Conventional normative modeling typically aims to infer the normative range of a phenotype, such as cortical thickness, for a fixed region of interest defined by a spatial query (Figure 1A). These approaches, referred to here as direct normative models, rely on predetermined spatial queries for each region under investigation. In contrast, SNM learns the normative distribution of a high-resolution phenotype using its graph spectral encoding on the eigenmode spatial basis set (Figure 1B). As a result, normative ranges can be rapidly computed across multiple spatial queries without refitting the model for each query (see Section 4.8 for details).

In this work, SNM utilizes connectivity eigenmodes derived from the random-walk Laplacian decomposition of high-resolution structural connectivity^35^ ($L_{rw}\psi_{i}=\lambda_{i}\psi_{i}$, see Section 4.7 Brain Signal Reconstruction). These eigenmodes, shown in Figure 1C, are ordered according to graph spectral frequencies, with higher frequencies capturing increasingly finer spatial details. Importantly, cortical thickness phenotypes exhibit a sparse cross-basis dependency structure when encoded on the eigenmode basis (Figure 1D). This sparsity contributes to the computational tractability of SNM, particularly when incorporating a larger number of eigenmodes.

### 2.2 Eigenmodes Reconstruct Cortical Thickness and Normative Query Maps

We first demonstrate the accuracy with which brain eigenmodes encode information represented across typical brain maps, including individual cortical thickness phenotypes, and three families of spatial queries commonly used in normative models. This tests the utility of eigenmodes as a low-dimensional basis for reconstructing brain maps, while also gauging the appropriate number of eigenmodes to be included in SNM.

**Figure. 2.**
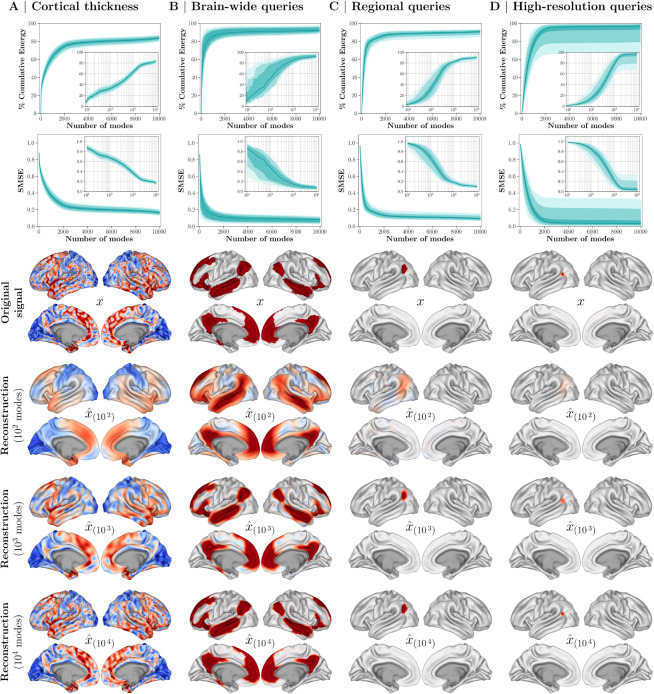
Cortical Signal Reconstruction Accuracy. The columns display eigenmode reconstruction accuracies for **(A)** individual participant’s cortical thickness phenotypes, as well as **(B)** brain-wide, **(C)** regional, and **(D)** high-resolution spatial queries. In the shaded line plots, the lines represent the median across all observations, while the shades indicate the [25, 75], [5, 95], and [1, 99] percentiles. For cortical thickness, the data comprises observations from 2,473 participants. For spatial queries, the data respectively includes a total of 25, 200, and 400 different brain-wide, regional, and high-resolution regions of interest. The first and second rows respectively show the cumulative energy and standardized mean square error (SMSE) as a function of the number of low-frequency eigenmodes used for spectral reconstruction (logarithmic x-axis for the insets). The third row illustrates one exemplary brain map from each category, while the last three rows show the same map reconstructed using 100, 1,000, and 10,000 eigenmodes, respectively. The cortical thickness projections (first column) display the thickness for a single exemplary individual.

As shown in Figure 2A, the reconstruction accuracy of individual cortical thickness phenotypes improves as more eigenmodes are included. While as few as 400 low-frequency modes (~ 0.7% of the total dimensions) capture 50% of the signal energy in cortical thickness maps, incremental inclusion of up to 2000 modes (~ 3.4% of the total) captures > 80% of signal energy, lowering reconstruction error to SMSE<0.2 (see Section on evaluation metrics). Improvements in accuracy beyond 2000 modes are marginal and plateau. Cortical projections of a participant’s thickness map visually demonstrate that low-frequency modes primarily capture spatially smooth, global features, while higher-frequency modes add finer spatial details to the reconstruction.

Similarly, eigenmodes parsimoniously reconstruct various types of spatial query maps used in multiscale normative modeling, including whole-brain, regional, and high-resolution queries. Whole-brain queries represent regions of interest spanning a large extent of the entire cortex, such as functional networks; regional queries correspond to parcels from a brain parcellation; and high-resolution queries are focused on a specific brain vertex (see Figure 2, and Section 4.9 Model Evaluation for details on these categories).

Fewer modes are needed to accurately reconstruct whole-brain maps than for regional or high-resolution maps. Including the first 100 modes (~ 0.2% of the total) captures around 52.6%, 33.3%, and 14.9% of the signal energy for whole-brain, regional, and high-resolution maps, respectively. By using 1000 modes (~ 1.7% of the total), approximately 85.5%, 82.1%, and 78.1% of the signal energy is captured for the respective query families, after which reconstruction error reduces to SMSE<0.2 and plateaus. Cortical projections confirm that including higher frequency modes (above K=1000) yields minimal visual improvements, mainly refining sharp transitions such as region borders. Supplementary visualizations are provided to further explore reconstruction residuals and assess the sensitivity of these findings to spatial granularity and cortical asymmetry (see Supplementary Figures S.12, S.5, S.8).

### 2.3 SNM Achieves Direct Model Accuracy with Adequate Modes

We leverage eigenmodes to approximate the normative ranges of any region of interest from its spectral encoding. SNM utilizes normative models trained on the spectral coefficients of cortical phenotypes (eigenmode loadings) and their cross-dependency structure to estimate the normative range for arbitrary normative queries (see Section 4.8.3 Spectral Normative Model (SNM) for methodological details). We evaluate SNM’s performance (with different numbers of modes: $k=\left\{10,10^{2},10^{3},10^{4}\right\}$) in reconstructing normative ranges for brain-wide, regional, and high-resolution thickness queries. These results are compared to a direct model trained on the exact cortical phenotype observed for each respective query.

Unlike the direct model, SNM relies solely on low-dimensional spectral approximations and is trained without prior information about the spatial extent of the query. Both models were trained on the same sample of 1,978 healthy individuals, covering a wide age range from 5 to 95 years (see Section 4.1 Brain Imaging Data and Supplementary Table S.1 for details). Model performance was assessed on an independent held-out sample of 495 individuals. Performance was evaluated using mean absolute error (MAE) to measure central tendency and mean standardized log-loss (MSLL) to assess the accuracy in modeling both normative means and deviations (see Section 4.9 Model Evaluation for details). Both models operate under the assumption that, after adjusting for covariate effects, the population distribution of each cortical thickness phenotype conforms to a Gaussian distribution; $y\;\sim \;𝒩\left(\mu_{y},\sigma_{y}^{2}\right)$ (see Section 4.8 Normative Modeling Framework).

**Figure. 3.**
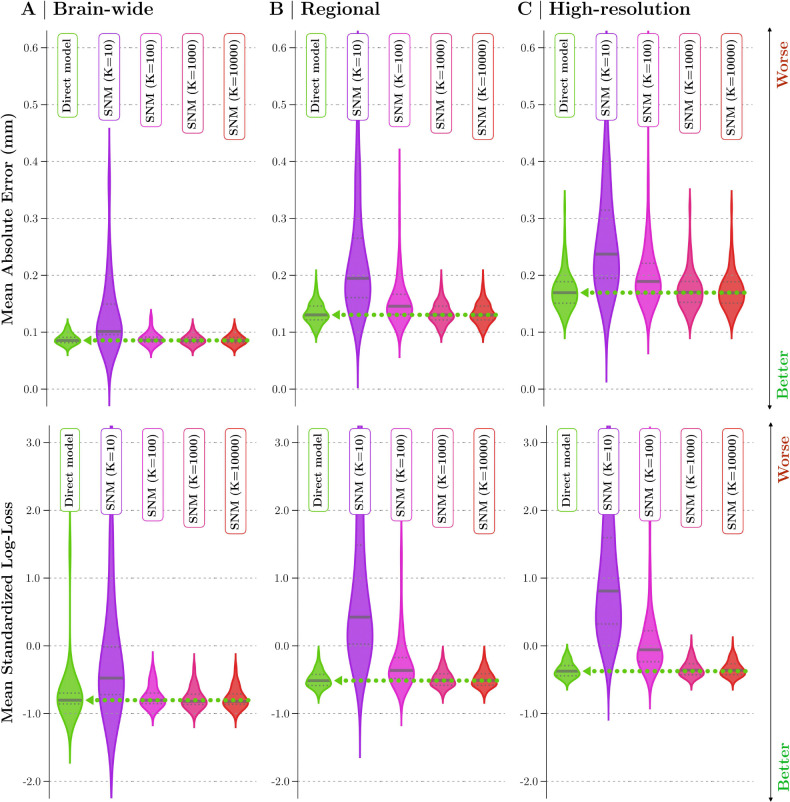
SNM Normative Performance. This figure compares performance metrics of the SNM at various number of modes ($k=10,10^{2},10^{3},10^{4}$) against a direct normative model. Performance is evaluated across three scales of **(A)** brain-wide, **(B)** regional, and **(C)** high-resolution spatial queries. The rows display performance in modeling the mean (mean absolute error, MAE, top row) and the overall shape of the normative distribution (mean standardized log-loss, MSLL, bottom row). Lower values indicate better performance for both metrics. Green violin plots represent the direct model (benchmark), and SNM performance is shown in shades from purple to red for different numbers of modes. The distributional variation in the violin plots illustrates the performance variability across different spatial queries within each spatial scale. Solid and dashed lines mark the median and first/third quartiles, with a green arrow denoting the direct model’s median for reference. In all evaluations, SNMs with at least 1000 modes achieve performance comparable to the direct model.

We evaluated the performance of SNMs with varying numbers of modes against direct models, testing both approaches across three spatial scales of normative queries: brain-wide, regional, and high-resolution. As shown in Figure 3, while SNM with only 10 modes shows substantially inferior performance across most queries, compared to the direct model. With as few as 100 modes, SNM yields valid normative estimates for the majority of brain-wide signals. However, most regional and high-resolution normative ranges require a higher number of modes for accurate normative estimation. Indeed, including at least 1000 modes in SNM (less than 2% of the total signal dimensionality) yields normative estimates that match the performance of direct normative models across all spatial scales. Adding more modes—up to 10,000—yields only marginal improvements, suggesting that the first ~ 1000 modes sufficiently capture the bulk of normative cortical thickness information. Supplementary analyses further explore the spatial distribution of SNM performance across brain regions, as well as the effects of query symmetry and granularity (see Supplementary Section A.3 Cortical projections of normative performance metrics and Supplementary Section A.4 Sensitivity Analyses). These findings underscore the robustness of SNM in reliably inferring normative ranges across different spatial scales, particularly when using at least 1000 modes.

### 2.4 SNM Enables High-Resolution Normative Brain Charting

**Figure. 4.**
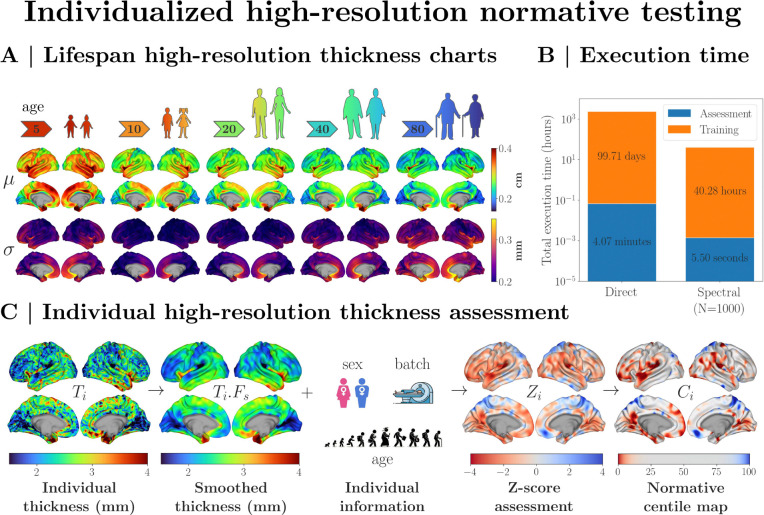
Application of Spectral Normative Assessments for Personalized High-resolution Normative Testing. **(A)** SNM can extract high-resolution lifespan charts of healthy cortical thickness changes. The model can provide estimates of normative vertex-wise thickness distribution moments (mean and deviation). **(B)** Execution times for training and assessments of SNM with 1,000 modes are compared to a hypothetical implementation using separate vertex-wise direct models. Times are displayed on a logarithmic scale due to the magnitude of differences; on a linear scale, the bar indicating SNM’s performance would be nearly imperceptible due to its significantly smaller execution time. **(C)** The high-resolution normative charts can be used for personalized assessments of individual brain scans. The cortical projections represent an exemplary individual from the test sample. Individual thickness values are smoothed using a selected kernel, and the high-resolution moments estimated by SNM are used to create individualized normative maps, indicating deviations quantified via high-resolution Z-scores or centile maps.

SNM not only achieves accuracy comparable to direct models but does so with significantly lower computational costs. This presents notable practical advantages, such as efficiently computing high-resolution normative ranges for cortical characteristics and deriving individualized normative estimates. Figure 4 highlights a practical application in which SNM is used to derive high-resolution normative ranges of cortical thickness and chart individual thickness deviations. By utilizing SNM with K=1000 modes, we derive normative estimates of cortical thickness across all cortical vertices using high-resolution queries centered on each respective vertex. This yields smooth high-resolution normative estimates of cortical thickness distribution (mean and standard deviation, as illustrated in Figure 4A), adjusted for age, sex, and site effects (see ???).

Notably, these estimates are computed in a fraction of the time required by the direct approach (Figure 4B). This efficiency improvement encompasses both steps of model training and assessment (i.e. generating normative centiles for a specific set of covariates). SNM significantly outperforms the direct model in terms of computational efficiency. Specifically, SNM eliminates the need to train separate models over tens of thousands of cortical vertices, a process that would otherwise require months to complete. Instead, by modeling the norms across the leading 1,000 eigenmode coefficients, the training time is reduced to less than 2 days. This similarly affects assessment times, reducing the time required to infer high-resolution ranges of a particular set of covariates (age, sex, and site) from minutes to seconds. Crucially, SNM can produce estimates for any arbitrary region of interest, a capability that is impossible with the direct model. As shown in Figure 4C, these high-resolution assessments enable the inference of personalized thickness deviations in individual participants, elucidating specific spatial patterns where cortical thickness deviates from healthy norms.

### 2.5 SNM Uncovers Cortical Signatures of Atrophy in Alzheimer’s Disease

Next, we demonstrate the practical utility of SNM by applying it to an independent clinical sample. Specifically, we analyze deviations in cortical thickness in an independent imaging dataset comprising three elderly cohorts of individuals with no cognitive impairment (healthy controls, HC, N=132), mild cognitive impairments (MCI, N=202), and Alzheimer’s Disease (AD, N=208) (see Section 4.1 Brain Imaging Data and Supplementary Section A.1.2 MACC Clinical Data for details about these cohorts). To leverage the transfer learning capabilities of SNM, we utilize the model trained on the large healthy dataset and fine-tune only the harmonization parameters. Consequently, the model is adapted to this independent dataset and can identify deviations in cortical thickness at high spatial resolution (i.e., vertex-wise z-scores, as described in the previous section).

**Figure. 5.**
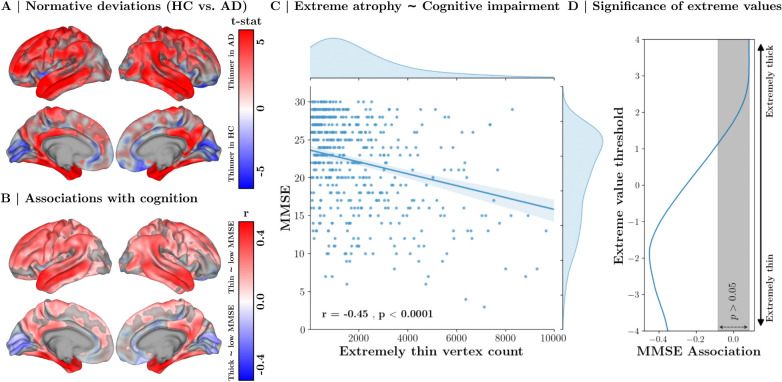
Cortical Signature of Atrophy in Alzheimer’s Disease and Its Cognitive Correlates. High-resolution deviation maps were used to compute the cortical signature of atrophy in an elderly clinical cohort and assess its ability to predict cognitive impairments that are associated with AD. **(A)** Group-level normative differences between HC and AD. **(B)** Vertexwise associations between normative deviation z-scores and cognitive performance (MMSE). **(C)** The ETVC metric, quantifying extreme atrophy, can predict cognitive performance in the clinical cohort. **(D)** Comparison of z-score thresholds for ETVC reveals that vertices with extreme atrophy provide the highest predictive power for cognitive impairment. Cortical projections highlight significant regions (dimmed for non-significant voxels) at α=5% after FDR correction. Abbreviations: HC: Healthy Cohort, AD: Alzheimer’s Disease, MMSE: Mini-Mental State Examination, ETVC: Extremely Thin Vertex Count, FDR: False Discovery Rate.

These deviation maps offer a powerful tool for investigating the normative thickness changes associated with AD. By spatially comparing deviation maps between healthy individuals and those diagnosed with AD, we uncover a cortical signature of structural atrophy in AD (Figure 5A). This demonstrates reduced cortical thickness in several neocortical regions spanning the temporal, parietal, and frontal lobes, indicating widespread atrophy associated with AD. While most abnormal differences indicate a thinner cortex in AD, we also find abnormally thick gray matter in certain visual areas along the lingual gyrus and cuneus. We also assess the relationship between cortical atrophy and cognitive impairment by examining Mini-Mental State Examination (MMSE) scores in relation to individualized normative assessment maps. Our results show significant effects linking cognitive impairment to widespread cortical thinning. These effects are highest in temporal regions but span several cortical areas (Figure 5B).

Findings of Figure 5A,B are consistent with previous studies and are therefore not claimed as a novel achievement of SNM but are reported to demonstrate its validity in replicating established findings. SNM can additionally generate individualized predictive cortical atrophy biomarkers through extreme value statistics. Specifically, we quantify the number of cortical vertices exhibiting extreme thinning for each individual (z<-1.96, see Section 4.10.2 Linking Atrophy to Cognitive Impairment for details). This extreme value statistic, termed Extremely Thin Vertex Count (ETVC), serves as a robust biomarker for cognitive impairment in AD. Namely, we find that individuals with more pronounced cortical thinning (higher ETVC) are at a significantly higher risk for cognitive impairment (r=-0.45,p<0.0001, Figure 5C).

Supplementary evaluations indicate that this association is reproducible within the AD cohort (r=-0.31,p<0.0001), but not within the healthy (r=-0.005,p=0.96) or MCI (r=-0.10,p=0.14) cohorts (see Supplementary Section A.6 Within-group Cognitive Associations). As shown in Figure 5D, we find that regions exhibiting extreme atrophy (-3<z<-2) contribute more significantly to the predictive power of ETVC biomarker compared to regions with mild atrophy (-2<z<0) or regions with hypertrophy (z>0). In supplementary analyses, we find an inferior predictive power for the normative z-score of mean cortical thickness compared to the high-resolution ETVC biomarker (see Section A.7 Brain-wide Cognitive Associations).

### 2.6 SNM Highlights Heterogeneity in Individual Cortical Atrophy

**Figure. 6.**
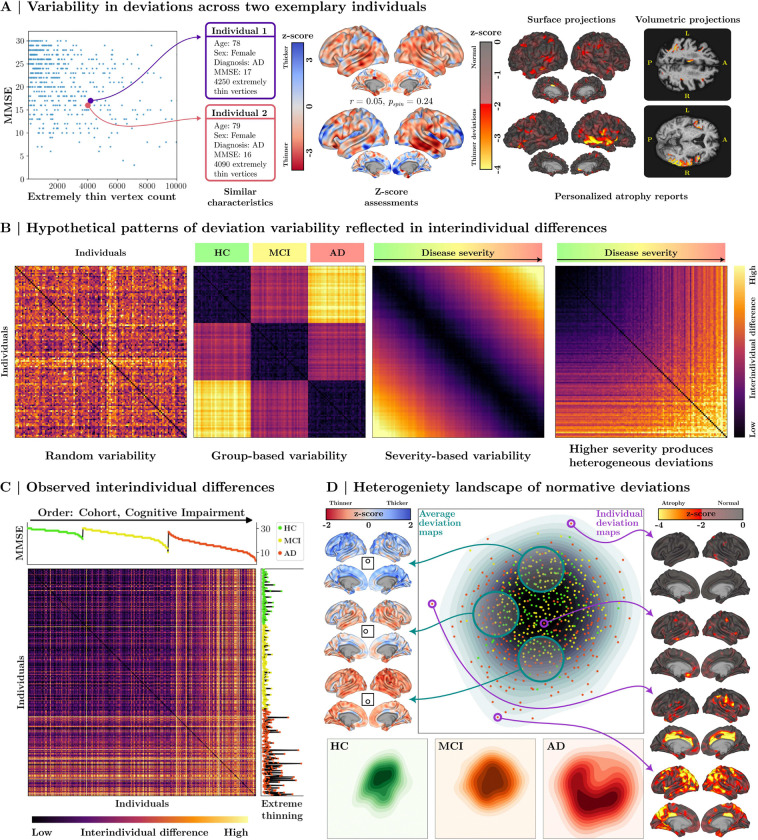
Heterogeneity Landscape of Atrophy Associated with Alzheimer’s Disease. Individualized high-resolution deviation maps were used to assess the extent of heterogeneity in AD-related normative deviations. **(A)** Examples of two individuals with similar age, sex, cognitive scores, and diagnosis, who nevertheless display markedly different patterns of cortical atrophy. These maps can be overlaid onto native scans as summary reports to assist assessments of cortical atrophy. **(B)** Interindividual differences in normative deviations are quantified using Euclidean distance between assessment maps. Four hypothetical heatmaps illustrate how the structure of interindividual differences depends on the underlying deviation mechanisms. Deviations can be completely random and unrelated to severity (left), strictly delineated by diagnostic groups (middle left), uniformly progressive across disease stages (middle right), or display heterogeneous divergence from norms (right). **(C)** An empirical interindividual difference matrix is computed from the clinical cohort’s deviation maps, with individuals sorted by clinical diagnosis and, within each diagnosis, by cognitive performance (from high to low MMSE). This distance matrix suggests that AD-diagnosed individuals exhibit heterogeneous normative deviations. **(D)** High-resolution normative assessments are projected onto a 2-dimensional landscape, while preserving interindividual difference structure. The central scatter plot displays the distribution of individuals from HC (green), MCI (yellow), and AD (red) cohorts within this landscape, with density plots (bottom) highlighting regions predominantly occupied by each cohort. Cortical projections (left) show average deviation maps for three exemplary local areas of this landscape, while exemplary individual deviation maps are shown (right) for four AD-diagnosed individuals.

While group-level analyses provide valuable insights into the average patterns of cortical atrophy associated with AD, they unavoidably obscure important interindividual variability. Group means can mask unique patterns of atrophy that are highly personalized, which may hold crucial implications for understanding disease mechanisms and tailoring clinical interventions. Complementing the group-level evaluations presented in the previous section, SNM enables the investigation of individualized deviation patterns. This facilitates a deeper exploration of the heterogeneity in cortical atrophy among individuals with AD. As illustrated in Figure 6A, two individuals with similar demographic characteristics, extreme thinning profiles, and identical diagnoses can exhibit markedly distinct cortical thinning patterns, with minimal spatial overlap ($r=0.05,\;p_{\mathrm{spin}}=0.24$). Importantly, SNM allows these deviation maps to be projected back into each individual’s native brain space, whether in surface or volumetric format, providing clinically interpretable atrophy maps that can potentially aid in personalized cortical atrophy assessments.

Differences in atrophy patterns may reflect distinct mechanisms through which pathology associates with normative deviations. To illustrate this, we simulated four hypothetical scenarios (Figure 6B), each showing differences between 60 individual normative deviation maps. Pairwise differences between maps were quantified by Euclidean distance to measure interindividual variability. In the first scenario, deviations occur in a random, non-systematic manner (Figure 6B, left). In the second scenario, deviations are tightly linked to diagnostic categories (Figure 6B, middle left, rows ordered by diagnosis group). In the third scenario, deviations are linked to disease severity, with individuals of similar severity exhibiting similar atrophy patterns (Figure 6B, middle right, rows ordered by severity). In the final scenario, increasing severity leads to greater heterogeneity in deviations (Figure 6B, right, rows ordered by severity).

We next compute the empirical interindividual difference matrix (Figure 6C) and sort it by diagnostic group (HC, MCI, and AD) and, within each group by cognitive performance (as a marker of symptom severity). We find that healthy controls and MCI patients exhibit relatively smaller differences in deviation patterns. However, individuals with AD show significantly greater variability, both when compared to healthy and MCI cohorts, as well as amongst themselves. These findings suggest that AD is associated with increasingly divergent cortical atrophy patterns across individuals, highlighting the heterogeneous nature of individual atrophy in AD.

To further elucidate the heterogeneity in deviation patterns, we embed the high-dimensional normative assessments into a lower-dimensional latent space that maintains the difference structure (Figure 6D, see Section 4.10.3 Examining Interindividual Heterogeneity for details). This reveals that healthy individuals form a dense central cluster, characterized by more homogeneous deviation maps. In contrast, the MCI group shows a more dispersed distribution, with greater variability in deviation patterns. The AD cohort exhibits the highest degree of deviation from the healthy cluster, with substantial diversity in the direction of deviations across individuals. This underscores various ways in which AD-diagnosed individuals deviate from normative cortical structure. When averaging deviation patterns within local neighborhoods of this landscape (cortical projections in Figure 6D, left), we observe distinct subgroups characterized by negligible (top), localized (middle), or widespread (bottom) atrophy (see Supplementary Section A.8 Heterogeneity Landscape Subgroups to further explore these local neighborhood projections). Exemplary cortical projections in Figure 6D (right) reiterate that AD-diagnosed individuals vary not only in the severity of cortical atrophy but also in the spatial distribution of these deviations.

## 3 Discussion

In this manuscript, we introduce SNM, a novel method to enhance the efficiency and versatility of normative brain charts. Our results demonstrate that SNM improves spatial specificity, reduces computational complexity, and maintains accuracy comparable to conventional, direct assessments across a broad spectrum of spatial normative queries. SNM shows considerable promise for precision brain charting, advancing the capabilities of existing approaches in modeling trajectories, analyzing group-level pathological deviations, and generating individualized profiles of brain anomaly characteristics.

### 3.1 Computational Efficiency

While direct models require re-training for each new query, SNM can provide accurate normative estimates for novel, a posteriori-defined queries without additional training. This results in significant time efficiency improvements. For example, as shown in Figure 4, high-resolution SNM assessments can be applied to different scales of spatial smoothing within seconds, without retraining the model. In contrast, direct models require retraining for every vertex when applying an alternative smoothing, a process that can take several months. SNM hence offers several orders of magnitude improvement in execution time, reducing it from months to seconds. This efficiency is particularly advantageous for computing vertex-wise normative maps and studying psychopathological deviations across large samples.

### 3.2 Spatial Versatility

Another key advantage of SNM is its ability to estimate a wide range of normative queries. The model can theoretically reconstruct an infinite number of queries through linear combinations of eigenmode basis functions, effectively spanning all signals that can be approximated by the low-pass graph filter. We demonstrate that this encompasses conventional normative queries used to chart cortical phenotypes. Additionally, our supplementary evaluations show that this extends to assessing other norms, such as cortical asymmetry, which can help detect abnormalities in cortical lateralization. Importantly, this means that a single pre-trained spectral normative model can generate regional normative charts for various brain parcellation schemes without additional cost. Given the multitude of existing brain atlases^36–45^, each offering particular advantages, SNM provides the flexibility to choose an atlas that best fits any specific research needs.

In addition to accommodating flexible regional queries, our results demonstrate that spectral normative models can generate high-resolution normative maps for smooth spatial queries, adhering to the low-pass reconstruction criterion. This means that if no regional brain atlas is preferred, the model can alternatively produce normative estimates at the resolution of acquired brain images (voxels or vertices). This is particularly useful for individual charting applications. By enabling efficient atlas-free normative assessment of individual brain characteristics, SNM provides high-resolution maps that pinpoint localized spatial deviations. This increased resolution can enhance the power and sensitivity to study heterogeneity in pathology-driven deviations from healthy norms^1^. Finally, this spatial versatility also enables on-demand assessment of individualized normative queries. For instance, by identifying patient-specific functional networks through individualized parcellation techniques^46^ and subsequently performing SNM on those individualized regional queries, we can directly assess whether particular behavioral differences are linked to underlying neuroanatomical deviations, offering a valuable tool for personalized clinical evaluation.

### 3.3 Accessibility Benefits

The concept of an a posteriori-defined query is particularly compelling, as it enables the use of largescale datasets while safeguarding the privacy of the original data sources. Conventionally, utilizing normative assessments for a user’s specific spatial query required access to an extensive set of imaging biobanks to train the normative model. Traditionally, performing normative assessments for arbitrary spatial queries required direct access to extensive imaging biobanks to train the normative model. In contrast, SNM decouples model training from query assessment, thereby eliminating the need for end-users to access the training data itself (see Figure 1).

Access to many large-scale imaging biobanks is subject to time-consuming data access procedures^47–50^. This limits the feasibility of community-wide normative modeling studies that require multiple datasets, as users may lack the necessary permissions to train the model from scratch. SNM overcomes this hurdle by enabling the creation of adaptable and openly accessible normative charts for brain development. Once a model is trained, posterior spectral estimates of normative moments can be shared, allowing assessments using the pre-trained model. This approach democratizes access to large-scale normative models and facilitates broader adoption in both research and clinical settings, particularly for end-users with limited data access.

As demonstrated by our evaluations on an independent clinical cohort, SNM can generalize effectively to previously unseen data from new sites, even with relatively small sample sizes, while appropriately modeling site-specific effects. This highlights SNM’s ability to leverage transfer learning, applying knowledge gained from large-scale cohorts to smaller datasets^51,52^. Furthermore, SNM’s architecture is well-suited for federated learning implementations, allowing the model to be trained on decentralized datasets across multiple sites^53^. These features position SNM as an efficient tool for studying clinical populations, enhancing the practical utility of normative modeling in situations where data collection and sharing is constrained.

### 3.4 Clinical Applications

In addition to systematic benchmarking and performance evaluations, we demonstrated SNM’s practical applications for high-resolution atrophy detection in a clinical cohort. Neurodegenerative mechanisms, such as amyloid-β plaque deposition and tau-related neurofibrillary tangles, are known to be associated with cortical atrophy in AD ^54–57^. Given that signs of cortical atrophy can emerge up to a decade before AD symptoms appear ^58,59^, SNM’s high-resolution assessments can facilitate early detection and characterization of neurodegeneration, enabling timely intervention. Our approach shows that a pre-trained SNM can produce individualized, high-resolution deviation maps, presenting a valuable biomarker for neurodegeneration that translates normative modeling insights into clinical applications^60^.

Our analysis supports previous findings by reproducing hallmark group-level cortical thinning patterns of AD, with the strongest reductions in cortical thickness centered on the temporal lobe (temporal pole, superior, middle, and inferior temporal gyri) ^57,61–66^. These effects also extend to regions of the neocortex, including the posterior cingulate gyrus ^64,67^, supramarginal and angular gyri ^61,62,64,65^, superior parietal lobule, superior, middle, and inferior frontal gyri ^61–64,68^, posterior cingulate cortex ^64,66,68^, and the precuneus ^57,61,62,64,68^. Additionally, other less established anomalies emerged, such as increased cortical thickness in certain visual and orbitofrontal areas^69^ that are potentially linked to compensatory neurodegenerative mechanisms^70^. Notably, our evaluations indicate that extreme atrophy quantified by ETVC at a higher threshold is a stronger predictor of cognitive impairment than mild atrophy, underscoring the utility of high-resolution normative assessment.

Our evaluations also emphasize the heterogeneity in individual cortical atrophy patterns, revealing diverse, pathology-driven deviation profiles. While group-level studies provide insights into common atrophy signatures, they may obscure individual-specific deviations^71^. This heterogeneity may reflect comorbidities^65,72–75^, which influence each person’s atrophy pattern across a varied landscape. Studies typically address this with clustering approaches to categorize pathological cohorts into common atrophy subtypes^64,66,76–80^; however, our findings reiterate that AD-related cortical deviations remain notably heterogeneous^81^.

Clustering methods, which group individuals into subtype patterns, may also obscure unique deviations when averaging patterns within subtypes, limiting the precision of subtype assignment for individuals^82^. We observed that some AD-diagnosed individuals exhibited negligible signs of atrophy, aligning with previous findings of subtypes characterized by minimal or no atrophy^56,64,66,79^. Conversely, some HC/MCI individuals showed significant atrophy without severe cognitive decline, possibly indicating markers of brain resilience^83,84^. These findings suggest that analyzing individual atrophy patterns along continuous principal or latent dimensions^85^ offers deeper insights into neurodegenerative variability than traditional subtype classifications, while also facilitating the study of resilience mechanisms in brain aging.

While these clinical findings underscore the potential of SNM for early detection and characterization of atrophy patterns, they remain preliminary. Future studies focused on specific clinical applications are necessary to fully assess SNM’s role as a diagnostic and prognostic aid in clinical settings and its broader potential in personalized medicine. For example, SNM could enhance diagnostic accuracy and aid in differential diagnosis, with deviation maps serving to distinguish between various types of dementia, such as Alzheimer’s disease, vascular dementia, Lewy body dementia, and frontotemporal dementia ^86^. Additionally, SNM shows promise as a prognostic tool; normative deviation maps in stroke or TBI patients could help evaluate the extent of damage to functional brain regions, allowing for more personalized rehabilitation plans and improved treatment monitoring. Finally, SNM’s heightened sensitivity to subtle, localized atrophy patterns may facilitate the detection of early biomarkers, such as microinfarcts, which often remain undetected but could identify individuals at risk, offering opportunities for preventive interventions before more severe events occur ^87^.

### 3.5 Future Directions and Limitations

The present evaluations demonstrate the feasibility of SNM as a flexible normative modeling framework with the potential for numerous applications in future research. An immediate next step could involve training SNM on a large sample of brain imaging scans, comprising hundreds of thousands of scans from multiple biobanks. This would support the development of a comprehensive, open-access database of spectral normative brain charts.

While the current study employs eigenmodes defined over the cortical surface mesh, future research could extend SNM to volumetric template spaces. By extracting voxel-wise brain eigenmodes^88^, SNM can also capture volumetric norms. Additionally, this framework could be applied to explore normative trajectories of high-resolution brain phenotypes across different modalities. For example, quantitative measures of high-resolution structural volume^13^, white matter microstructure^89–91^, functional organization^92,93^, or metabolic processes quantified by Positron Emission Tomography^94^ could serve as alternative normative phenotypes of interest. Such explorations would contribute to a deeper, multifaceted understanding of healthy brain development and organization.

Several limitations warrant consideration. First, the current spectral model assumes Gaussianity, modeling phenotypes as normally distributed across the population. This model does not extend to phenotypes with substantial skewness or kurtosis. Although handling non-Gaussian distributions is beyond the scope of this paper, this limitation could be addressed in future work by integrating SNM with recent advancements in likelihood warping techniques^16,91^ to model non-Gaussian distributions.

Second, SNM assumes that, with an adequate number of low-frequency eigenmodes, the phenomenon under evaluation (e.g., cortical thickness) is well-captured by the spectral basis set. While our study has demonstrated this for cortical thickness (Figure 2), the utility of this approach for other modalities and phenotypes depends on whether this assumption holds true. Similarly, the spatial query under study should be restricted to a low-pass graph spectral regime. Future applications of this method for different phenotypes or novel queries should include tests of reconstruction accuracy to ensure the validity of the spectral basis set for those specific contexts.

Lastly, this study utilized a spatial basis set constructed from connectome eigenmodes via singular value decomposition of the random walk Laplacian shift operator. However, spectral normative modeling can be applied using various orthonormal basis sets. For example, eigenmodes can be derived from different brain features (e.g., geometry^95^, diffusion^25,27^, or function^96,97^) or other shift operators (e.g. combinatorial Laplacian, symmetric normalized Laplacian, or the fractional Laplacian)^98,99^. We chose to use connectome eigenmodes due to their ease of extension across both cortical hemispheres while aligning with anatomical and geometrical brain landmarks, and the random walk Laplacian shift operator for its smoothness and robustness to degree distribution ^100^. Nevertheless, it should be noted that SNM is theoretically generalizable to any orthogonal basis set for information reconstruction and is not limited to brain eigenmodes.

## 4 Methods

In the ensuing sections, we first describe the brain imaging datasets utilized in this work. We provide a formal description of the high-resolution cortical thickness phenotypes used as input to the normative modeling framework. Next, we introduce the mathematical foundations of SNM, covering core concepts from graph signal processing and normative modeling. Finally, we describe experiments undertaken to evaluate SNM’s performance and suitability.

### 4.1 Brain Imaging Data

This study uses three publicly available healthy brain imaging datasets that were provided and curated by the Human Connectome Project (HCP)^101^; namely, HCP’s Young Adult^102^, Aging^103^, and Development^104^ cohorts. These cohorts collectively comprised 2473 healthy individuals (54.7% female) spanning a wide age range, from 5 to 100 years old. The imaging acquisition and preprocessing followed previously established pipelines and were broadly consistent across the three cohorts^105,106^. In addition to the healthy datasets used to train the normative model, we evaluated the model’s translational capabilities to real-world data from memory clinics (Memory, Ageing & Cognition Centre at the National University of Singapore: MACC)^107,108^. This data comprised 542 samples (ages 50–91, 61.4% female), including 132 cognitively healthy individuals (24.3%), 202 individuals with mild cognitive impairment (MCI; 37.3%), and 208 individuals diagnosed with Alzheimer’s Disease (38.4%) based on DSM-IV criteria^108^. Across all datasets, FreeSurfer processing of the T1-weighted (T1w) structural brain imaging data was used to extract high-resolution maps of cortical thickness. Additional details regarding acquisition and preprocessing procedures for each dataset are provided in the Supplementary Information (see Section A.1 Image Acquisition and Preprocessing).

### 4.2 Train Test Split

Healthy individuals were stratified into train and test splits to enable validation of the fitted models on unseen data. Namely, 80% ($N_{\mathrm{train}}=1978$ individuals) of the healthy data were used to train the model, and the remaining 20% ($N_{\mathrm{test}}=495$) were used for out-of-sample validation ($N_{p}=N_{\mathrm{train}}+N_{\mathrm{test}}$). A single split fold was used, with a randomized approach that controlled for covariate distributions of age, sex, and dataset by stratification; this ensured that the covariates had similar distributions across splits^109^. Supplementary Figure S.1 provides a visual summary of covariate distributions before and after splitting.

### 4.3 Cortical Thickness

For all cohorts, cortical thickness estimates for each vertex comprising the cortical surface mesh was sourced from preprocessed data. HCP’s minimal processing pipeline^105^ contains procedures utilizing FreeSurfer outputs^110^ to provide high-resolution thickness estimates for the fs-LR 32k surface mesh template. This template surface uses 32,492 vertices to model each hemisphere, resulting in an average inter-vertex distance of approximately 2 mm; it includes a total of 59,412 vertices after the exclusion of the medial wall. Vertices are aligned between the left and right hemispheres, enabling inter-hemispheric comparison)^102^. The same transformation was used to project MACC sample’s FreeSurer cortical thickness estimates to the fs-LR surface space.

In this space, cortical thickness is represented as a high-resolution vector $T_{i}\in R^{1\times N_{v}}\left(N_{v}=59,\;412\right)$, providing a thickness estimate for each vertex of the i th individual’s cortical surface. The collection of these high-resolution thickness features across all participants forms the complete high-resolution thickness data sample $T\in R^{N_{p}\times N_{v}}$ which is used to estimate and evaluate normative ranges of thickness variation across the human lifespan. These data are divided into a training sample $T_{\mathrm{train}}\in R^{N_{\mathrm{train}}\times N_{v}}$ used to construct the normative model and an independent test sample $T_{\mathrm{test}}\in R^{N_{\mathrm{test}}\times N_{v}}$ used to evaluate the normative model’s goodness of fit.

### 4.4 Normative Covariates

In normative modeling, the aim is to provide a statistical model that describes the distribution of a variable of interest y with respect to a set of covariates C (fixed effects), while potentially accounting for batch effect Z (random effects)^111^. For simplicity, we consider the covariates of age and sex, although other covariates can be straightforwardly incorporated. Moreover, batch effects were included in the model to facilitate harmonization among the three imaging cohorts (development, young adult, and aging).

### 4.5 Clinical Fine-tuning

The normative model, pre-trained on $N_{\mathrm{train}}$ healthy individuals, was subsequently fine-tuned for the clinical sample of $N_{MACC}=674$ individuals using transfer learning. During fine-tuning, the model parameters related to age and sex were kept frozen to maintain consistency with the initial healthy cohort model. However, site parameters for dataset harmonization were fine-tuned on a subsample of 66 healthy individuals (50% of the HC cohort) from the MACC clinical cohort. The fine-tuned model was then applied to the rest of the clinical sample to generate individualized, high-resolution normative deviation maps. These maps were utilized in normative evaluations of the clinical cohort.

### 4.6 High-resolution Connectomes

Our proposed approach uses brain connectivity eigenmodes computed from a reference brain connectivity structure^23^. Connectivity eigenmodes were selected for their ability to span both cortical hemispheres, enabling a cohesive representation of interhemispheric relationships. In contrast, geometric modes analyze hemispheres separately, overlooking interhemispheric interactions. Additionally, as many brain disorders with structural deviations from normative patterns are hypothesized to express spatial signatures aligned with anatomical connectivity, connectome eigenmodes are optimally suited to capture such abnormalities^28–33^. High-resolution structural connectivity data were utilized for this purpose^35^.

To this end, we sourced connectomes computed from a tractography pipeline detailed elsewhere^112^. In brief, diffusion-weighted imaging data were used to estimate structural connectivity. Probabilistic tractography was conducted using MRtrix3 to reconstruct whole-brain tractograms (5 million streamlines, with anatomically constrained tractography). Tractography endpoints were used to construct connectomes encoding streamline count at the resolution of the fs-LR 32k template (same space as the thickness information, with $N_{v}=59,\;412$ vertices representing network nodes). Individual connectomes were combined to form an estimate of group-level connectivity. Connectome spatial smoothing was performed to account for endpoint inaccuracies and improve connectome reliability^113,114^. This yielded a high-resolution weighted adjacency matrix $A\in R_{+}^{N_{v}\times N_{v}}$ in which element $A_{i,j}$ denotes the group average strength of structural connectivity between nodes i and j. The eigenmodes resulting from this high-resolution connectome mapping pipeline were previously tested to assess their accuracy in encoding brain signals^23^.

### 4.7 Brain Signal Reconstruction

Graph signal processing enables the study of data encoded on a graph/network structure^115^. As such, considering that the human brain is fundamentally a network structure, graph signal processing can be utilized to design new ways to analyze and study the brain^116^. Here, we utilize dimensionality reduction and signal reconstruction techniques that encode cortical information in a lower-dimensional latent space^117,118^. To this end, we use high-resolution connectomes as a weighted graph 𝒢:(𝒱,A) where $𝒱=\left\{1,\;2,\cdots ,N_{v}\right\}$ is the set of high-resolution cortical nodes (vertices) and $A\in R_{+}^{N_{v}\times N_{v}}$ is a weighted (non-negative) adjacency matrix. We utilize GSP to model any brain signal $x\in R^{N_{v}\times 1}$ that is defined on the set of vertices 𝒱. In this context, we model vertex-wise cortical thickness estimates as a brain signal.

The random-walk Laplacian matrix $L_{rw}=I-D^{-1}A$ is used as the graph shift operator (where $D\in R^{N_{v}\times N_{v}}$ denotes the diagonal strength matrix). This shift operator is diagonalized via singular value decomposition $L_{rw}=\Psi \Lambda \Psi^{-1}$ to compute an orthogonal basis for information reconstruction. Here, $\Psi \in R^{N_{v}\times N_{v}}$ denotes the full set of eigenvectors (left singular vectors, also referred to as eigenmodes), and $\Lambda \in R^{N_{v}\times N_{v}}$ denotes the diagonal matrix of eigenvalues (singular values) associated with the eigenmodes such that $\Lambda_{i,i}=\lambda_{i}$ is the i th smallest eigenvalue and $\Psi_{i}\in R^{N_{v}\times 1}\;(i$ th column of Ψ) refers to its associated eigenmode. For any brain signal $x\in R^{N_{v}\times 1}$, a graph Fourier transform (GFT) can encode the signal in graph spectral domain $\tilde{x}=\Psi^{T}x$, where $\tilde{x}\in R^{N_{v}\times 1}$ denotes the encoded signal, where ${\tilde{x}}_{i}$ is the i th element of $\tilde{x}$ which quantifies the loading of the brain signal on the i th eigenmode (${\tilde{x}}_{i}=\Psi_{i}^{T}x$). Conceptually, this encoding transforms the brain signal x defined in the spatial domain to a latent embedding $\tilde{x}$ in graph spectral domain. The encoded signal in the graph spectral domain can be mapped back to the spatial domain via the inverse GFT: $x=\Psi \tilde{x}=\Psi \Psi^{T}x=Ix$. Notably, this transformation, utilizing the full set of eigenmodes, yields an exact reconstruction of any brain signal. Our goal is to use only a fraction of Ψ to derive a low-dimensional representation of brain signals. We selected the random-walk Laplacian shift operator as it yields eigenmodes encoding topological frequency such that eigenmodes associated with smaller eigenvalues capture signals that vary more smoothly along network edges^100^.

Deriving the full set of eigenmodes ($\Psi_{1}$ to $\Psi_{N_{v}}$) is computationally demanding; however, graph Fourier filtering enables efficient approximation of brain signals using a filter that only requires a limited set of eigenmodes. Specifically, we can define a diagonal filtering matrix $H\in R^{N_{v}\times N_{v}}$, where $H_{i,i}=h\left(\lambda_{i}\right)$ denotes the filter’s frequency response to the eigenmode $\Psi_{i}$ associated with the frequency $\lambda_{i}$. An ideal low-pass filter $H_{\left(k\right)}$ with frequency response $h_{k}$ that discards the information of eigenmodes with graph frequencies higher than $\lambda_{k}$ is defined using the step function:

(1) $$H_{{\left(k\right)}_{i,i}}=h_{k}\left(\lambda_{i}\right)$$

(2) $$h_{k}(\lambda )=\left\{\begin{array}{ll} 1 & \text{if}\;\lambda \leq \lambda_{k}\\ 0 & \text{otherwise} \end{array}\right.$$

Using this low-pass filter, brain signal x is approximated by $x\approx {\overset{ˆ}{x}}_{\left(k\right)}=\Psi H_{\left(k\right)}\Psi^{T}x=\Psi H_{\left(k\right)}\tilde{x}$, where ${\overset{ˆ}{x}}_{\left(k\right)}$ is the low-pass filtered approximation of x reconstructed from the first k eigenmodes. It should be noted that all elements beyond the k th row and column of $H_{\left(k\right)}$ are zeros. As a result, this approximation does not require knowledge about higher frequency eigenmodes $\Psi_{i}:i>k$. Specifically, let ${\overset{ˆ}{\Psi }}_{\left(k\right)}\in R^{N_{v}\times k}$ denote the set of first k eigenmodes (i.e. first k columns of Ψ); the low-pass approximation of brain signal x can be achieved as follows:

(3) $${\overset{ˆ}{x}}_{\left(k\right)}={\overset{ˆ}{\Psi }}_{\left(k\right)}{\overset{ˆ}{\Psi }}_{\left(k\right)}^{T}x={\overset{ˆ}{\Psi }}_{\left(k\right)}{\tilde{x}}_{\left(k\right)}$$

Where ${\tilde{x}}_{\left(k\right)}\in R^{k\times 1}$ is the k-dimensional encoding of the signal based on the first k eigenmodes in the graph spectral domain. As such, low-pass graph frequency filtering effectively reduces an $N_{v}$-dimensional brain signal x to a k-dimensional latent graph frequency representation. We anticipate that the majority of spatial variation in a brain signal such as cortical thickness is captured by a low-pass approximation of adequate bandwidth (i.e. an appropriate choice of k). This provides a tool to control the trade-off between computational complexity (dimensionality) and spatial specificity. Choosing a lower value for k reduces the computational demand but increases simplification and lowers spatial specificity. Selecting a k that gives an optimal balance between computational tractability and spatial specificity is thus important.

### 4.8 Normative Modeling Framework

In the context of our paper, conventional normative models encompass all univariate approaches in which the normative ranges of a fixed spatial query (e.g., the thickness of a region of interest) are modeled as a function of demographic covariates. SNM is a general framework that is compatible with alternative normative models, such as GAMLSS^2,119^, BLR^17,120^, and HBR^15,111^, etc.). For clarity and without loss of generality, we focus on an exemplary instantiation using Hierarchical Bayesian Regression (HBR)^15^. We begin by formally defining a spatial normative query to specify the region of interest, followed by an overview of HBR (referred to as the direct method, see Figure 1A). We then describe how SNM extends the direct approach by using eigenmodes, facilitating the estimation of arbitrary, a posteriori-defined spatial normative queries.

#### 4.8.1 Spatial Normative Query

The concept of a spatial normative query is new to this work. A researcher may wish to determine, or *query*, a normative range over a specific spatial extent, such as an individual vertex on the cortical mesh, a cortical region or hemisphere, or the entire brain. A spatial normative query denotes a brain signal $x\in R^{N_{v}\times 1}$ that is the result of such a query. Typically, this query characterizes averaging the phenotype over the spatial region of interest, although other descriptive statistics could be applied. For instance, if we want to determine the normative range for cortical thickness averaged over the entire cortex, the spatial query would store the associated weights for the cortex-wide averaging operation, i.e. $x=1_{N_{v}}\cdot \frac{1}{N_{v}}$ where $1_{n}$ denotes a unit vector of length $n(1_{n}=(1,\;1,\cdots ,1{)}^{T}\in R^{n\times 1})$. Similarly, if we are interested in modeling the average thickness of an arbitrary brain region with $N_{r}$ vertices, the respective spatial query x can be defined as follows ($x_{i}$ denotes the i th element/row of $x={\left(x_{1},x_{2},\cdots ,x_{N_{v}}\right)}^{T}$):

(4) $$x_{i}=\left\{\begin{array}{ll} \frac{1}{N_{r}} & \text{if}\;\text{vertex}\;v_{i}\;\text{belongs}\;\text{to}\;\text{the}\;\text{region}\;\text{of}\;\text{interest}\\ 0 & \text{otherwise} \end{array}\right.$$

Our definition of a spatial query provides significant flexibility to establish reference ranges across arbitrarily defined regions and networks. For instance, the normative thickness of a functional network can be evaluated using a spatial query with the weights of x proportional to the probability of a vertex belonging to that network (such that $\sum_{i\in \left\{1,2,\cdots ,N_{v}\right\}}\;x_{i}=1$). Moreover, this notion enables the assessment of comparative normative questions. For instance, we can assess the difference in average thickness of the left and right hemispheres to assess cortical asymmetry/lateralization:

(5) $$x_{i}=\left\{\begin{array}{ll} \frac{1}{N_{\mathrm{left}}} & \text{if}\;v_{i}\;\text{belongs}\;\text{to}\;\text{the}\;\mathrm{left}\;\text{hemisphere}\\ \frac{-1}{N_{\mathrm{right}}} & \text{if}\;v_{i}\;\text{belongs}\;\text{to}\;\text{the}\;\text{right}\;\text{hemisphere} \end{array}\right.$$

where $N_{\mathrm{left}},\;N_{\mathrm{right}}$ respectively denote the number of vertices in each hemisphere.

#### 4.8.2 Direct Method

The direct method refers to the conventional normative modeling approach in which a fixed a-priori-defined spatial query is used (e.g. mean thickness). In this manuscript, we utilize HBR as the normative approach used for the direct method^15,111^. Using a fixed spatial query x, the direct method models the expression of the phenotype (e.g. cortical thickness) on the query in the training set, $y\in R^{N_{\mathrm{train}}\times 1}$; which is computed as the inner product between the spatial query x and the high-resolution thickness data $T,\;y=T_{\mathrm{train}}.x$. HBR assumes that these observations follow a normal distribution $y\;\sim \;𝒩\left(\mu_{y},\sigma_{y}^{2}\right)$ and aims to model its moments (mean and variance) as a function of covariates (age and sex) while controlling for batch effects:

(6) $$\mu_{y}=f_{\mu }(\mathrm{age},\;\mathrm{sex},\;\mathrm{batch})=w_{\mu }\Phi (\mathrm{age})+\alpha_{\mu }(\mathrm{sex})+\beta_{\mu }(\mathrm{batch})+\epsilon_{\mu }$$

(7) $$\sigma_{y}=f_{\sigma }^{+}(\mathrm{age},\;\mathrm{sex},\;\mathrm{batch})=w_{\sigma }^{+}\Phi (\mathrm{age})+\alpha_{\sigma }^{+}(\mathrm{sex})+\beta_{\sigma }^{+}(\mathrm{batch})+\epsilon_{\sigma }$$

In particular, $w_{\mu }$ and $w_{\sigma }^{+}$ are weights that model the fixed effect of age by a basis expansion Φ. A B-spline basis expansion (cubic spline with three evenly spaced knots; df=5) was used to capture the non-linear effects of age. The fixed effect of sex is modeled by intercepts for mean ($\alpha_{\mu }$) and deviation ($\alpha_{\sigma }^{+}$). Batch effects (for dataset harmonization) are modeled as intercepts for the mean ($\beta_{\mu }\;\sim \;𝒩(\mu_{\beta_{\mu }},\sigma_{\beta_{\mu }})$) and deviation ${(\beta }_{\sigma }^{+}\;\sim \;𝒩(\mu_{\beta_{\sigma }^{+}},\sigma_{\beta_{\sigma }^{+}}^{2}))$ that are randomly drawn from another prior distribution; this results in hierarchically modeling batch as a random effect. The prior assumptions of the Bayesian model can be summarized as follows:

(8) $$w_{\mu }(i)\;\sim \;𝒩(0,\sigma_{w_{\mu }(i)}^{2}),\;\alpha_{\mu }\;\sim \;𝒩\left(0,\sigma_{\alpha_{\mu }}^{2}\right),\;\beta_{\mu }\;\sim \;𝒩(\mu_{\beta_{\mu }},\sigma_{\beta_{\mu }}^{2})$$

(9) $$w_{\sigma }^{+}(i)\;\sim \;{𝒩}^{+}(0,\sigma_{w_{\sigma }^{+}(i)}^{2}),\;\alpha_{\sigma }^{+}\;\sim \;{𝒩}^{+}(0,\sigma_{\alpha_{\sigma }^{+}}^{2}),\;\beta_{\sigma }\;\sim \;{𝒩}^{+}(\mu_{\beta_{\sigma }},\sigma_{\beta_{\sigma }}^{2})$$

Where ${𝒩}^{+}$ denotes a positive half-normal distribution. The random effect parameters are hierarchically sampled from a set of batch hyperparameters:

(10) $$\mu_{\beta_{\mu }}\;\sim \;𝒩(0,\sigma_{\mu_{\beta_{\mu }}}^{2}),\;\sigma_{\beta_{\mu }}\;\sim \;{𝒩}^{+}(0,\sigma_{\sigma_{\beta_{\mu }}}^{2}),\;\mu_{\beta_{\sigma }}\;\sim \;{𝒩}^{+}(0,\sigma_{\mu_{\beta_{\sigma }}}^{2}),\;\sigma_{\beta_{\sigma }}\;\sim \;{𝒩}^{+}(0,\sigma_{\sigma_{\beta_{\sigma }}}^{2})$$

Notably, the model can quantify the normative deviation of an individual subject $y_{i}$ using a Z-score:

(11) $$z=\frac{y_{i}\;-\;\mu_{y}}{\sigma_{y}}$$

This Z-score can be used to compute normative centiles using the cumulative distribution function (CDF) of the normal distribution.

#### 4.8.3 Spectral Normative Model (SNM)

Next, we explain the theory behind the proposed SNM framework. In the training phase, k direct normative models are independently fitted to the projection of phenotypes on the first k brain eigenmodes with the lowest graph frequencies. This forms the low-dimensional latent approximation containing information about the low-frequency characteristics of the cortical phenotype. For example, in the case of cortical thickness, for each i≤k, the spectral coefficient $s_{i}\in R^{N_{train}\times 1}$ associated with $\Psi_{i}$ is first computed:

(12) $$s_{i}=T_{\mathrm{train}}\;.\Psi_{i}$$

Similar to the direct model, normative estimators of the mean ($f_{\mu_{i}}$) and deviation $\left(f_{\sigma \;i}^{+}\right)$ are trained to model normative ranges of all low-frequency spectral coefficients:

(13) $$\forall i\leq k:s_{i}\;\sim \;𝒩\left(\mu_{s_{i}},\sigma_{s_{i}}^{2}\right)\Rightarrow \left\{\begin{array}{l} \mu_{s_{i}}=f_{\mu_{i}}(\mathrm{age},\;\mathrm{sex},\;\mathrm{batch})\\ \sigma_{s_{i}}=f_{\sigma \;i}^{+}(\mathrm{age},\;\mathrm{sex},\;\mathrm{batch}) \end{array}\right.$$

The normative model for each spectral coefficient is the same as the direct case (see equations 6, 7, 8, 9, 10). Next, the normative ranges of any arbitrary spatial query is approximated using this set of k pre-trained normative models. For any spatial query x, its observed cortical phenotype $y=T_{\mathrm{train}}\;.x$ is approximated by a low-pass graph filter (see equation 3):

(14) $$y=T_{\mathrm{train}\;}.x\approx {\overset{ˆ}{y}}_{\left(k\right)}=T_{\mathrm{train}}\cdot {\overset{ˆ}{x}}_{\left(k\right)}\;\;=T_{\mathrm{train}}{\overset{ˆ}{\Psi }}_{\left(k\right)}{\overset{ˆ}{\Psi }}_{\left(k\right)}^{T}x=T_{\mathrm{train}}{\overset{ˆ}{\Psi }}_{\left(k\right)}{\tilde{x}}_{\left(k\right)}\;\;=\sum_{i\leq k}T_{\mathrm{train}}\Psi_{i}{\tilde{x}}_{i}=\sum_{i\leq k}\;s_{i}{\tilde{x}}_{i}$$

The variable of interest y can hence be approximated by a linear combination of the spectral coefficients $s_{i}$. Notably, if the spatial query is frequency-bounded this approximation will be exact (i.e. if the query is a linear combination of low-frequency eigenmodes such that ${\tilde{x}}_{i}=0$ for i>k). Equation 14 suggests that for any spatial query x, the associated phenotype y can be modeled as a multivariate normal distribution such that its normative ranges are estimated by the linear combination $y=\sum_{i\leq k}s_{i}{\tilde{x}}_{i}$. This is formalized by the following equation:

(15) $$\forall x\in R^{N_{v}\times 1},y=T_{\mathrm{train}}\cdot x\;:\;y\;\sim \;𝒩\left(\mu_{y},\sigma_{y}^{2}\right)\Rightarrow \left\{\begin{array}{l} \mu_{y}\approx g_{\mu }(F_{\mu_{\left(k\right)}},{\tilde{x}}_{\left(k\right)})\\ \sigma_{y}\approx g_{\sigma }^{+}\left(\Sigma ,{\tilde{x}}_{\left(k\right)}\right) \end{array}\right.$$

Where $g_{\mu }$ and $g_{\sigma }^{+}$ are functions that respectively approximate the mean and standard deviation of the multivariate normal distribution based on the trained low-pass spectral moments ($F_{\mu_{\left(k\right)}},\Sigma$). Explicitly, $g_{\mu }$ defines the mean of the normal distribution y as a linear combination of the means of each spectral coefficient and is based on the graph spectral encoding ${\tilde{x}}_{\left(k\right)}$:

(16) $$g_{\mu }(F_{\mu_{\left(k\right)}},\;{\tilde{x}}_{\left(k\right)})=\sum_{i\leq k}\;f_{\mu_{i}}{\tilde{x}}_{i}={\left(f_{\mu_{1}},\;f_{\mu_{2}},\;\cdots ,\;f_{\mu_{k}}\right)}_{1\times k}\;.\;{\;\tilde{x}}_{\left(k\right)}=F_{\mu_{\left(k\right)}}\cdot {\tilde{x}}_{\left(k\right)}$$

where $F_{\mu_{\left(k\right)}}$ is used to summarize the set of estimated means for the first k spectral coefficients ($y_{1},\cdots ,y_{k}$) into a 1×k vector. Note that equation 16 arises because the expected value of a linear combination of a set of random variables is equal to the linear combination of their respective expected values:

(17) $$E\left[a_{1}X_{1}+a_{2}X_{2}+\ldots +a_{k}X_{k}\right]=a_{1}E\left[X_{1}\right]+a_{2}E\left[X_{2}\right]+\ldots +a_{k}E\left[X_{k}\right]$$

Moreover, $g_{\sigma }^{+}$ defines the standard deviation of the normal distribution y as a function of the covariance matrix describing cross-basis dependencies of spectral coefficients $\Sigma \in R_{+}^{k\times k}$:

(18) $$g_{\sigma }^{+}=\sqrt{\sum_{i,j\leq k}{\tilde{x}}_{i}{\tilde{x}}_{j}\Sigma_{i,j}}=\sqrt{{\tilde{x}}_{\left(k\right)}^{T}\Sigma {\tilde{x}}_{\left(k\right)}}$$

Equation 18 describes how the standard deviation of the variable of interest y is related to the covariance matrix Σ describing dependencies across spectral coefficients $s_{i}$. This equation is based on the following notion that relates the variance of a linear combination of a set of normally distributed variables to their covariance structure:

(19) $$Var\left(\sum_{i=1}^{k}a_{i}X_{i}\right)=\left(\sum_{i=1}^{k}a_{i}^{2}Var\left(X_{i}\right)\right)+\left(\sum_{i=1}^{k}\sum_{j=1(j\neq i)}^{k}\;a_{i}a_{j}Cov\left(X_{i},X_{j}\right)\right)$$

Elements of Σ can also change as a function of covariates and batch effects. We note that for a pair of random variables (X and Y), covariance (Cov(X,Y)) and correlation (Corr(X,Y)) are related by $Cov(X,Y)=\sigma_{X}\sigma_{Y}Corr(X,Y)$, where σ denotes the standard deviation of each variable. We use this notion to separate the effect of within-mode variance from the effect of cross-mode correlation on Σ. In particular, the following equation decomposes the cross-basis covariance matrix Σ into a correlation matrix P and an independent set of eigenmode standard deviations:

(20) $$\Sigma =diag\left(F_{\sigma (k)}^{+}\right)\cdot P\cdot diag\left(F_{\sigma (k)}^{+}\right)$$

where $F_{\sigma (k)}^{+}$ summarizes the set of estimated standard deviations of the first k spectral coefficients $\left(y_{1},\cdots ,y_{k}\right)$ to a 1×k vector, and the diag() operator is used to map a 1×k vector to a k×k diagonal matrix:

(21) $$diag\left(F_{\sigma (k)}^{+}\right)=\left(\begin{array}{cccc} f_{\sigma \;1}^{+}, & 0, & \cdots , & 0\\ 0, & f_{\sigma \;2}^{+}, & \cdots , & 0\\ \vdots , & \vdots , & \ddots , & \vdots \\ 0, & 0, & \cdots , & f_{\sigma \;k}^{+} \end{array}\right)$$

Thus far, we show that any spatial query can be represented as a multivariate normal distribution across k spectral components, the normative ranges of which can be approximated from the mean ($F_{\mu_{\left(k\right)}}$, standard deviation ($F_{\sigma \;(k)}^{+}$), and cross-correlation (P) of spectral coefficients. Mean and standard deviation estimates are computed similarly to a direct HBR model. However, approximations may be needed to estimate the cross-correlation structure. This is because the complexity of the correlation structure increases quadratically with the number of eigenmodes included in the low-pass approximation (k). We hence impose a sparsity constraint on the correlation structure using a threshold (ρ=0.25) on the observed cross-correlation of spectral phenotypes within the training sample. Our evaluations indicate that the cross-correlation matrix is naturally sparse (see Figure 1D). As a result, the majority of elements $P_{i,j}\;:\;Corr\left(s_{i},s_{j}\right)\leq \rho$ are small and will hence be replaced with zero through this sparsification step. The remaining pairs of suprathreshold correlations form a sparse matrix representation as summarized by the following derivation of P:

(22) $$P_{i,j}=\left\{\begin{array}{ll} 0 & \text{if}\;Corr\left(s_{i},s_{j}\right)\leq \rho \\ \rho_{s_{i},s_{j}} & \text{if}\;Corr\left(s_{i},s_{j}\right)>\rho \end{array}\right.$$

The suprathreshold elements $\rho_{s_{i},s_{j}}$ are affected by covariates (age and sex) and batch effects. As such, for every suprathreshold element, the spectral coefficient pair was modeled as a bivariate normal distribution:

(23) $$\left[\begin{array}{l} s_{i}\\ s_{j} \end{array}\right]\;\sim \;𝒩\left(\left[\begin{array}{l} \mu_{s_{i}}\\ \mu_{s_{j}} \end{array}\right],\left[\begin{array}{cc} \sigma_{s_{i}}^{2} & \rho_{s_{i},s_{j}}\sigma_{s_{i}}\sigma_{s_{j}}\\ \rho_{s_{i},s_{j}}\sigma_{s_{i}}\sigma_{s_{j}} & \sigma_{s_{j}}^{2} \end{array}\right]\right)\Rightarrow \left\{\begin{array}{l} \text{constants}\left\{\begin{array}{c} \mu_{s_{i}}=f_{\mu_{i}};{\;\mu }_{s_{j}}=f_{\mu_{j}}\\ \sigma_{s_{i}}=f_{\sigma i}^{+};{\;\sigma }_{s_{j}}=f_{\sigma \;j}^{+} \end{array}\right.\\ \rho_{s_{i},s_{j}}=tanh\left(f_{\rho_{i,j}}(\mathrm{age},\;\mathrm{sex},\;\mathrm{batch})\right) \end{array}\right.$$

where spectral coefficients’ estimates of the mean ($\mu_{s_{i}},\mu_{s_{j}}$) and deviation ($\sigma_{s_{i}},\sigma_{s_{j}}$) were inputted as constants (yielded from the solution of Equation 13). The cross-correlation estimate ($\rho_{s_{i},s_{j}}$) was adjusted by a hyperbolic tangent function to limit the range of possible correlation values to [-1,1]. Effects of covariates and batch were modeled as follows:

(24) $$f_{\rho_{i,j}}(age,\;\mathrm{sex},\;\mathrm{batch})=w_{\rho }\Phi (\mathrm{age})+\alpha_{\rho }(\mathrm{sex})+\beta_{\rho }(batch)+\epsilon_{\rho }$$

with the following prior assumptions:

(25) $$w_{\rho }(i)\;\sim \;𝒩(0,\sigma_{w_{\rho }(i)}^{2})\;,\;\alpha_{\rho }\;\sim \;𝒩\left(0,\sigma_{\alpha_{\rho }}^{2}\right)\;,\;\beta_{\rho }\;\sim \;𝒩(\mu_{\beta_{\rho }},\sigma_{\beta_{\rho }}^{2})$$

and the following hierarchical batch priors:

(26) $$\mu_{\beta_{\rho }}\;\sim \;𝒩(0,\sigma_{\mu_{\beta \rho }}^{2}),\;\sigma_{\beta_{\rho }}\;\sim \;{𝒩}^{+}(0,\sigma_{\sigma_{\beta \rho }}^{2})$$

### 4.9 Model Evaluation

We use three different families of spatial normative queries (x) to evaluate SNM’s performance. These families include (I) *brain-wide*, (II) *regional*, and (III) *high-resolution* signals, each of which groups queries according to their spatial scale. Brain-wide queries are regional masks that describe coarse and large-scale characteristics along the cortex. Namely, this includes a total of 25 queries that represent the average of total cortical thickness, as well as the average thickness over predefined brain networks that divide the brain into 7/17 functionally distinct segments (Yeo functional networks)^39^. Regional queries included 200 signals, each describing the average cortical thickness within a region of interest as defined by a brain parcellation. For regional queries, we used a homotopic parcellation of the cortex comprising 200 cortical regions^121^ (Yan200 atlas). In the supplementary analysis, we evaluate the sensitivity of our findings to atlas granularity (see Supplementary Figures S.5, S.6). Finally, a total of 400 high-resolution signals were constructed, each of which describes the average cortical thickness in the vicinity of a specific cortical vertex. In this case, we first randomly select one vertex from every region of the Yan400 cortical parcellation^121^ and compute an 8mm FWHM smoothing kernel based on the geodesic distance to that vertex^114^. The smoothing kernel serves two key purposes: (1) to mitigate the impact of local intersubject registration misalignments and (2) to ensure vertex-resolution queries are appropriately reconstructed by the low-pass graph filter, thereby reducing ringing effects in reconstructions. In a supplementary analysis, we evaluate the sensitivity of our evaluations to the smoothing kernel choice (see Supplementary Figures S.5, S.7). The signals evaluated here quantify the average thickness of the cortex with different spatial granularities (ranging from tens of centimeters to a few millimeters). While such average queries are the only type of normative queries that have been previously studied for brain charting, in supplementary analyses we evaluate the applicability of our proposed model to other possible cases, e.g. when a comparative asymmetric norm is being studied (see Supplementary Figures S.8, S.9, S.10, and S.11).

For all signal families (whether presented in the main text or supplementary information), we evaluated the performance of SNM (using different values of k) based on (i) reconstruction accuracy, normative model’s (ii) goodness of fit of the central tendency, and (iii) total goodness of fit of the estimated distribution. These evaluation criteria are detailed in the ensuing sections.

#### 4.9.1 Reconstruction Accuracy

For the spectral approach to provide accurate estimates of normative ranges, both the cortical phenotype under study (i.e. cortical thickness) and the spatial normative query must be adequately reconstructed by the low-dimensional latent space defined by the brain eigenmodes. We hence use the following two metrics to evaluate the validity of the brain eigenmodes for reconstructing brain signals:

##### Energy Proportion:

For any arbitrary brain signal $x\in R^{N_{v}\times 1}$ with graph spectral encoding $\tilde{x}=\Psi^{T}x$, the total energy of the signal can be quantified by $E(x)=\sum_{i\in \left\{0\cdots N_{v}\right\}}x_{i}^{2}=\sum_{i\in \left\{0\cdots N_{v}\right\}}{\tilde{x}}_{i}^{2}=E(\tilde{x})$. In other words, the signal energy in vertex domain is equal to the signal energy in the spectral domain (Parseval’s theorem^122^). As such, the proportional contribution of every single eigenmode $\Psi_{i}$ to the total signal energy can be quantified by ${\tilde{x}}_{i}^{2}/E(x)$. We provide a cumulative line plot of observed ranges for this proportional energy for different brain signals. This indicates the number of eigenmodes that capture major energy proportions for different brain signals.

##### Standardized Mean Squared Error (SMSE):

This measure normalizes the mean squared error (MSE) by signal variance to provide an error metric that does not depend on signal variance^123^. Using this metric, a trivial reconstruction that replaces values of all vertices $x_{i}$ with the signal mean will have an SMSE of one, and a perfect reconstruction will result in an SMSE of zero. By considering ${\overset{ˆ}{x}}_{\left(k\right)}$ to be a reconstruction of the original brain signal x, we compute SMSE for different ks to quantify reconstruction error at different low-pass frequency bandwidths. A high SMSE (close to one) indicates poor reconstruction performance and a low SMSE (close to zero) marks accurate reconstruction.

#### 4.9.2 Model’s Central Tendency

Independent of how accurately the eigenmode basis reconstructs various brain signals, we also need to validate that SNM yields accurate normative fits for spatial queries. This is quantified by measures of goodness of fit of the central tendency^2^. These measures can particularly indicate the accuracy of the normative model in explaining trends in the data. To this end, we utilize the mean absolute error (MAE) of model predictions. An out-of-sample MAE score was evaluated using 20% of the data in the test set. Specifically, for any spatial query x, the thickness of that query in the test set is measured $y\in R^{N_{\mathrm{test}}\times 1}=T_{\mathrm{test}}$. x and MAE quantifies the extent to which these thickness values deviate from model predictions $MAE=E[\left|y-\mu_{y}\right|]=\sum |y_{i}-\mu_{y_{i}}|/N_{\mathrm{test}}$. MAE was computed for each of the three families of spatial queries, comparing the performance of a direct HBR model against four alternative SNMs, which utilized the first 10, 100, 1,000, and 10,000 eigenmodes, respectively. Since all models provided mean thickness estimates in millimeters, MAE offers a practical measure of the average error in millimeters when inferring the thickness of a given spatial query.

#### 4.9.3 Model’s Total Goodness of Fit

Measures of central tendency, such as mean or median, assess only the accuracy of modeling normative trends but do not capture the validity of the estimated distribution’s deviations. To evaluate the model’s overall goodness of fit—including both mean and variance estimates—we utilize the mean standardized log loss (MSLL)^123^. Mean log loss quantifies the mean negative log probability of the observed data (in the test sample) under the hypothesis of the fitted normative model:

(27) $$log-loss(\text{model})=-\frac{1}{N_{\mathrm{test}}}log\left(p\left(y|\mu_{y},\sigma_{y}\right)\right)=-\frac{1}{N_{\mathrm{test}}}\sum_{i=1}^{N_{\mathrm{test}}}\left(\frac{1}{2}log\left(2\pi \sigma_{y_{i}}^{2}\right)+\frac{{\left(y_{i}\;-\;\mu_{y_{i}}\right)}^{2}}{2\sigma_{y_{i}}^{2}}\right)$$

MSLL is computed by comparing the log loss of competing models against the log loss of a trivial reference model that is based on the mean and standard deviation of the training sample, MSLL=log-loss(model)-log-loss(reference). By definition, the trivial model achieves an MSLL of zero, and models providing better than trivial normative ranges will yield negative MSLL values. Here, we estimate log loss using a censored log-likelihood measure to reduce the sensitivity to outliers^2,124^. As such, for observations falling outside of the range of 1^st^ to 99^th^ percentile, the value of log loss was replaced by -log(0.02). This censored MSLL score favors models with accurate distribution estimates within this percentile range and is thus robust against extreme outliers.

### 4.10 Clinical Evaluations

In Section 2.5 SNM Uncovers Cortical Signatures of Atrophy in Alzheimer’s Disease and Section 2.6 SNM Highlights Heterogeneity in Individual Cortical Atrophy, we examined the clinical insights provided by SNM when applied to the clinical MACC dataset. A subset of 66 healthy individuals from the MACC cohort was used to fine-tune the pretrained SNM for the clinical sample. During fine-tuning, parameters linking cortical phenotypes to age and sex ($w_{\mu },\;w_{\sigma }^{+},\;w_{\rho },\;\alpha_{\mu },\;\alpha_{\sigma }^{+},\;\alpha_{\rho }$) were held constant, while only site-specific parameters for batch effect harmonization ($\beta_{\mu },\;\beta_{\sigma }^{+},\;\beta_{\rho }$) were updated based on the MACC data. The fine-tuned model was then applied to generate normative deviation maps for smoothed, vertex-resolution cortical thickness phenotypes.

#### 4.10.1 HC vs. AD Comparison

A two-sample t-test was conducted to compare high-resolution deviation maps between the healthy and AD groups within the MACC dataset. To identify vertices with statistically significant differences, a nonparametric false discovery rate (FDR) correction^125^ was applied across all vertices (FDR-α=5%, 1000 permutations), controlling for multiple comparisons. Vertices surpassing the corrected threshold were deemed to exhibit significantly different deviation patterns between groups.

#### 4.10.2 Linking Atrophy to Cognitive Impairment

We next evaluated the potential of normative deviation maps to serve as biomarkers of cognitive impairment. To begin, we performed a univariate vertex-wise assessment, measuring the Pearson correlation coefficient between normative z-scores and cognitive performance scores (MMSE). The significance of associations was evaluated with a nonparametric FDR correction via permutation testing. Specifically, we shuffled MMSE scores across 1000 permutations, calculating correlation coefficients for each permutation to produce a null distribution under the hypothesis of no association between normative deviation and cognitive performance. Using this null distribution, we derived nonparametric p-values for each vertex, which were subsequently FDR-corrected at α=5%.

In addition, we derived an extreme value statistic^89^ for atrophy by counting vertices with a z-score below a threshold of −1.96 for each individual. This measure, termed Extremely Thin Vertex Count (ETVC), was then tested for its predictive power on MMSE scores via Pearson correlation. To assess the sensitivity of ETVC to the threshold choice, we repeated this analysis for a range of z-score thresholds. We performed these assessments within each cohort—HC, MCI, and AD—to evaluate the specificity of ETVC as a biomarker for AD-related cognitive impairment (see Supplementary Section A.6 Within-group Cognitive Associations).

#### 4.10.3 Examining Interindividual Heterogeneity

Next, we aimed to demonstrate the capabilities of the SNM in revealing the heterogeneity landscape of brain atrophy in AD. We quantified interindividual differences in atrophy by computing the Euclidean distance between vectors of vertex-level z-score deviation maps, yielding a $N_{MACC}\times N_{MACC}$ distance matrix that captures interindividual differences. This matrix was visualized as a heatmap to provide an overview of the variation across individuals. Additionally, we applied multidimensional scaling (MDS) ^126^ to reduce the distance matrix to a 2-dimensional embedding, providing a spatial representation of interindividual differences. Average deviation maps for subgroups within local vicinities in this 2-dimensional space were then visualized as cortical projections (see Figure 6 and Supplementary Section A.8 Heterogeneity Landscape Subgroups). These visualizations, along with the original heatmap, illustrate that AD is associated with heterogeneous atrophy patterns: while individuals with AD deviate from healthy norms, they do not conform to a single or even multiple stereotypical patterns of deviation.

## Supplementary Material

Supplement 1

## Data Availability

The code for implementing, evaluating, and generalizing the SNM framework to independent cohorts will be made openly available in the following Git repository: [Link to the repository will be added here.] This repository will also host the fitted parameters of the lifespan SNM model. Analyses involving the healthy lifespan sample were conducted using publicly available data from the Human Connectome Project, accessible through the Connectome Coordination Facility (www.humanconnectome.org). The clinical AD dataset can be obtained via a data-transfer agreement with the MACC (http://www.macc.sg/).
