# Supplementary material for: Spectral normative modeling of brain structure
Source: medRxiv. 2025 Jan 21:2025.01.16.25320639. Preprint. [Version 1] doi: 10.1101/2025.01.16.25320639 (PMC11838943; doi:10.1101/2025.01.16.25320639)
Supplement: Supplement 1 [file NIHPP2025.01.16.25320639v1-supplement-1.pdf]

medRxiv preprint doi: https://doi.org/10.1101/2025.01.16.25320639; this version posted January 21, 2025. The copyright holder for this preprint (which was not certified by peer review) is the author/funder, who has granted medRxiv a license to display the preprint in perpetuity.
It is made available under a CC-BY 4.0 International license .

Supplementary material for:

Spectral normative modeling of brain structure

Mansour L., S. et al. (2025)

A Supplementary Information

A.1 Image Acquisition and Preprocessing

A.1.1 HCP Lifespan Data

Healthy human brain imaging data from three cohorts of the Human Connectome Project (HCP) was aggregated to encompass a broad age range spanning the human lifespan. Table S.1 summarizes the population demographics across datasets and their respective train-test splits. The following paragraphs detail each cohort's image acquisition protocols and preprocessing steps.

Cohort	HCP-D		HCP-YA		HCP-A	
Split	Train	Test	Train	Test	Train	Test
Sample Size	521	131	877	219	580	145
Age	14.41 ± 4.06	14.53 ± 4.07	28.79 ± 3.69	28.78 ± 3.70	60.29 ± 15.70	60.61 ± 15.89
Sex	53.7% female	54.2% female	54.4% female	54.3% female	55.9% female	56.6% female
Average Thickness (mm)	3.01 ± 0.14	3.03 ± 0.14	2.64 ± 0.08	2.65 ± 0.08	2.57 ± 0.15	2.56 ± 0.17

Table S.1. Sample Characteristic of HCP lifespan dataset. Note: Values presented as $\mu \pm \sigma$ indicate the mean (μ) and standard deviation (σ).

HCP Young Adult Data Young adult imaging data was sourced from the HCP S1200 release[102] (HCP-YA). This contained structural MRI images obtained from 1096 healthy participants (54.4% female) aged 22 to 37 years old. A comprehensive report on imaging acquisition and preprocessing is available elsewhere[105]. In brief, the images were acquired with a Siemens 3T Skyra scanner with a 32-channel head coil (housed at Washington University). T1w images were acquired using a single-echo MPRAGE sequence (0.7mm isotropic resolution, TR = 2400ms, TE = 2.14ms, TI = 1000ms, flip angle = 8°, no partial Fourier)[105]. Acquired images underwent preprocessing using the HCP's minimal preprocessing pipeline. Notably, structural images underwent gradient distortion correction, skull stripping, readout distortion correction, and bias field correction, followed by a nonlinear registration to a standard template volumetric space. Cortical surface reconstruction was performed using FreeSurfer (version 5.2) which produced 3-dimensional meshes for the white and pial cortical surface along with segmentation and folding-based registration to the *fsaverage* surface. Finally, the outputs

medRxiv preprint doi: https://doi.org/10.1101/2025.01.16.25320639; this version posted January 21, 2025. The copyright holder for this preprint (which was not certified by peer review) is the author/funder, who has granted medRxiv a license to display the preprint in perpetuity.
It is made available under a CC-BY 4.0 International license .

were registered to the *Conte69* surface template with a multimodal surface matching algorithm (MSMAll)[138] and downsampled to triangulated meshes with ~32k vertices per cortical hemisphere, i.e. the *fs-LR* 32k space.

HCP Aging Data The aging imaging data was sourced from the HCP-Aging Lifespan 2.0 release[103] (HCP-A) which included structural MRI images from 725 healthy adults (56% female) aged 36 to 100+ years old. For all participants aged above 90 years old, age was reported as 100 to avoid releasing protected health information. The acquisition and preprocessing protocols for this cohort were generally consistent with the earlier acquired HCP-YA protocols, with particular necessary changes due to hardware differences that are detailed elsewhere[106]. In summary, data were collected on Siemens 3T Prisma scanners (with 32-channel head coils) located at 4 different acquisition sites in the United States. Particularly, T1w images were collected by a multi-echo MPRAGE sequence with real-time motion correction (0.8mm isotropic voxels, TR = 2500ms, TE = 1.8/3.6/5.4/7.2ms, TI = 1000ms, flip angle = 8°, 6/8 slice partial Fourier)[106]. The preprocessing pipeline for structural images was similar to that of HCP-YA following the minimal preprocessing pipeline[105] with minor variations including a different version of FreeSurfer (version 6.0) and an update to MSMAll using higher order smoothness constraints[139].

HCP Development Data The development imaging data was sourced from HCP-Development Lifespan 2.0 release[104] (HCP-D) which contained structural MRI images of 652 healthy participants (53.8% female) aged 5 to 22 years old. This cohort aimed to adapt existing HCP protocols to the practical challenges of studying developmental populations. The acquisition and processing of the HCP-D dataset[104] were generally consistent with the HCP-A dataset[103]. Namely, similar Prisma scanners (with 32-channel head coils) were used to collect imaging data across four acquisition sites, while a tailored 32-channel head coil was used for 5-7 year-old participants[104,106]. Identical to HCP-A, a multi-echo MPRAGE sequence was used to collect T1w images followed by the same preprocessing procedures[106].

A.1.2 MACC Clinical Data

The clinical data (part of MACC harmonization study) was acquired by the Memory, Ageing & Cognition Centre at the National University of Singapore[107,108]. The dataset includes 542 participants aged 50 to 91 years (61.4% female, see Table S.2 for detail), grouped into three cohorts: i) a healthy cohort (HC) of 132 cognitively healthy individuals, ii) a mild cognitive impairment cohort (MCI) of 202 individuals with cognitive impairment but no AD, and iii) an AD cohort of 208 individuals with an AD diagnosis according to the Diagnostic and Statistical Manual of Mental Disorders, Fourth Edition (DSM-IV) criteria. AD diagnosis followed internationally accepted criteria using the National Institute of Neurological and Communicative Disorders and Stroke and Alzheimer's Disease and Related Disorders Association (NINCDS-ADRDA) guidelines.

Each participant underwent neuroimaging and comprehensive clinical and neuropsychological evaluation, including the Mini-Mental State Examination (MMSE). MRI was performed on a 3T Siemens Magnetom Trio Tim scanner with a 32-channel head coil at the Clinical Imaging Research Centre of the National University of Singapore. The imaging protocol included a T1-weighted MPRAGE sequence (1 mm isotropic resolution, TR = 2300 ms, TE = 1.9 ms, TI = 900 ms, flip angle = 9°, 192 sagittal slices, matrix size = 256 × 256)[140]. T1-weighted scans were preprocessed using FreeSurfer 6.0 `recon-all` pipeline to generate cortical surfaces and thickness estimates in the fsnative surface space [141,142]. Matlab scripts and commands from Connectome Workbench and FreeSurfer were then used to project thickness data to the fs-LR surface space, aligning it with HCP thickness data to enable transfer learning from HCP datasets to MACC.

medRxiv preprint doi: https://doi.org/10.1101/2025.01.16.25320639; this version posted January 21, 2025. The copyright holder for this preprint (which was not certified by peer review) is the author/funder, who has granted medRxiv a license to display the preprint in perpetuity.
It is made available under a CC-BY 4.0 International license .

Cohort	HC	MCI	AD
Description	No cognitive impairment	Cognitive impairment no dementia	Diagnosis of AD
Sample Size	132	202	208
Age	68.49 ± 7.58	74.29 ± 6.70	75.49 ± 7.19
Sex	54.5% female	58.4% female	68.8% female
MMSE	27.39 ± 1.90	24.04 ± 3.88	15.60 ± 4.96

Table S.2. Sample Characteristic of MACC-H dataset. Note: Values presented as $\mu \pm \sigma$ indicate the mean (μ) and standard deviation (σ).

A.2 Stratified Sample Splitting

As described in the Methods section, train test splitting was performed by a randomized approach that controls for the distribution of sample covariates (age, sec, and dataset/batch). In particular, this was implemented by `scikit-learn`'s `train_test_split` function by utilizing the `stratify` parameter. First, continuous values for individual age were grouped into 20 bins by Quantile-based discretization. Next, discrete values encoding age, sex, and dataset information were utilized for stratified splitting. Supplementary Figure S.1 demonstrates the stability of covariate distribution after train test splitting.

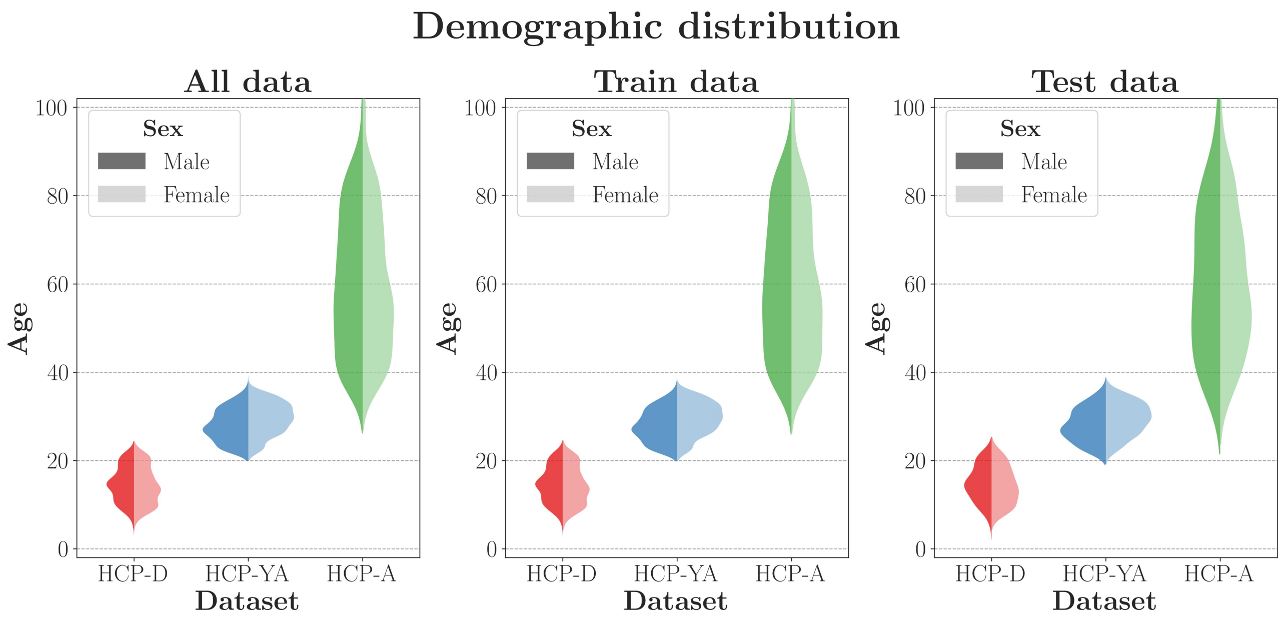

Supplementary Figure. S.1. Distribution of demographic information before and after splitting. The distribution of demographic information is displayed for the entire sample before splitting (left) and after splitting into training (center) and test (right) sets. The violin plots illustrate the age distribution (y-axis) across different datasets (x-axis). Each plot differentiates between male (dark shades) and female (bright shades) participants. The stratified group splitting ensures that the demographic distributions of covariates are matched between the training and test samples.

A.3 Cortical projections of normative performance metrics

Figure 3 presents a summary report comparing SNM performance with that of the direct model. In the following sections, we expand upon these findings, detailing the regional distribution of performance metrics to offer deeper insights into comparative SNM evaluations.

A.3.1 Brain-wide normative estimates

Figure S.2 presents the performance of various SNMs in reconstructing brain-wide thickness maps, compared to a direct model. The direct model is trained on observed cortical phenotypes for each

medRxiv preprint doi: https://doi.org/10.1101/2025.01.16.25320639; this version posted January 21, 2025. The copyright holder for this preprint (which was not certified by peer review) is the author/funder, who has granted medRxiv a license to display the preprint in perpetuity.
It is made available under a CC-BY 4.0 International license .

spatial query ($y = T_{train}\ .\ x$), while SNMs rely solely on spectral approximations ($y \approx \sum_{i \leq k} s_i \tilde{x}_i$). Four SNMs are evaluated, which respectively utilize the first $k = \{10, 10^2, 10^3, 10^4\}$ eigenmodes.

With as few as 10 modes, spectral normative models can produce acceptable normative estimates for some brain-wide signals, indicated by negative MSLL values. However, specific brain-wide signals, such as the average thickness of the ventral attention network and the limbic network (from Yeo's 7 functional networks[39]), exhibit poor normative estimates at this level, showing noticeably inferior MAE and MSLL scores compared to the direct model. However, including at least 100 modes in the spectral normative model yields performance comparable to the direct model. These findings were also extended in sensitivity analysis for brain-wide asymmetric queries (see Supplementary Figure S.9).

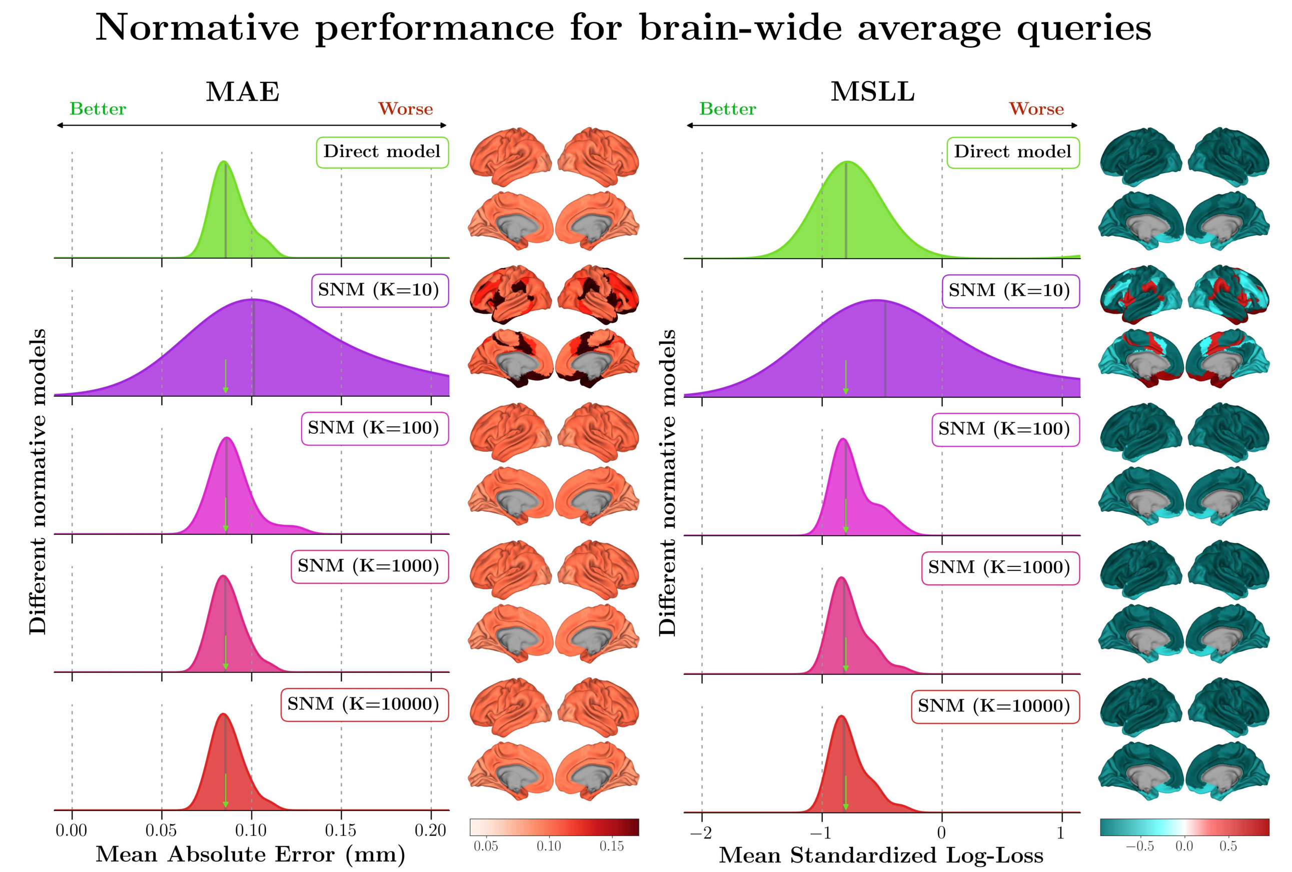

Supplementary Figure. S.2. Comparison of performance for different normative models assessing brain-wide spatial queries. Normative performance is evaluated using out-of-sample assessments of direct and spectral normative models. The top row displays the performance of the direct model, while the next four rows show the performance of spectral models with 10, 100, 1,000, and 10,000 eigenmodes, respectively. Mean absolute error (MAE, left) and mean standardized log-loss (MSLL, right) are used to quantify model performance. Violin plots illustrate the distribution of performance measures across 25 different brain-wide signals, including cortex-wide average thickness and thickness averaged over 7 and 17 functional networks. Median values are indicated by gray lines on the violin plots. A green arrow marks the median performance of the direct model over spectral performance distributions for visual comparison. Cortical surface projections show normative performance values for 7 functional networks, with shared colorbars across all five rows to facilitate visual comparison.

A.3.2 Regional normative estimates

Figure S.3 extends the previous comparison to regional signals. At this level of spatial specificity, SNM with only 10 modes shows substantially inferior performance across several brain regions. Including the first 100 modes results in acceptable performance for most brain regions, except for regions on the post-central gyrus, insular cortex, and orbitofrontal cortex. Incorporating up to 1000 modes in the spectral model achieves performance matching that of the direct model across all brain regions. Further inclusion of modes (up to 10,000) provides minimal additional benefits in goodness of fit, as measured by MAE or MSLL. Sensitivity analyses evaluate the robustness of these findings to regional granularity and signal asymmetry (see Supplementary Figures S.6, S.10).

medRxiv preprint doi: https://doi.org/10.1101/2025.01.16.25320639; this version posted January 21, 2025. The copyright holder for this preprint (which was not certified by peer review) is the author/funder, who has granted medRxiv a license to display the preprint in perpetuity.
It is made available under a CC-BY 4.0 International license .

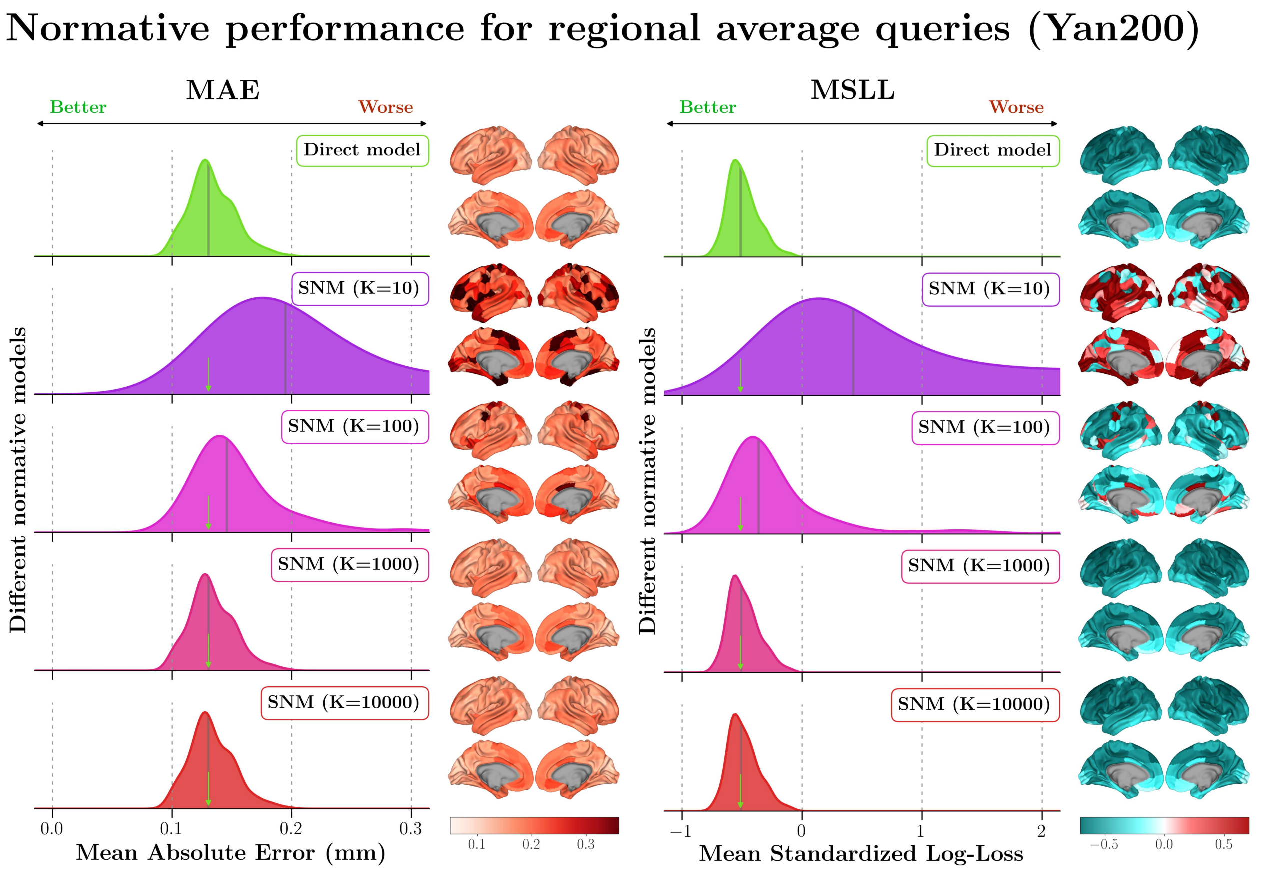

Supplementary Figure. S.3. Comparison of performance for different normative models assessing regional spatial queries. Normative performance is evaluated using out-of-sample assessments of both direct and spectral normative models. The top row shows the performance of the direct model, while the following four rows present the performance of spectral models with 10, 100, 1,000, and 10,000 eigenmodes, respectively. Mean absolute error (MAE, left) and mean standardized log-loss (MSLL, right) are used to quantify model performance. Violin plots display the distribution of performance measures across 200 different spatial queries, representing regional average thickness based on the Yan200 atlas. Median values are marked by gray lines on the violin plots. A green arrow marks the median performance of the direct model over spectral performance distributions for visual comparison. Cortical surface projections show normative performance values for each region, with shared colorbars across all five rows to aid visual comparison.

A.3.3 High-resolution normative estimates

Finally, Figure S.4 assesses SNM's performance in estimating high-resolution normative charts for spatial queries centered on various cortical surface voxels. Given the computational infeasibility of applying the direct model to every vertex, we evaluated a randomly selected subset of 400 vertices across the cortical surface. At this level of spatial specificity, spectral normative models with 10 and 100 modes fail to estimate normative ranges effectively compared to the direct model. However, the spectral model with 1000 modes achieves performance comparable to the direct model. Further inclusion of 10,000 modes yields marginal performance improvements. We also evaluated the sensitivity of these results to spatial granularity and asymmetry in normative queries (see Supplementary Figures S.7, S.11).

medRxiv preprint doi: https://doi.org/10.1101/2025.01.16.25320639; this version posted January 21, 2025. The copyright holder for this preprint (which was not certified by peer review) is the author/funder, who has granted medRxiv a license to display the preprint in perpetuity.
It is made available under a CC-BY 4.0 International license .

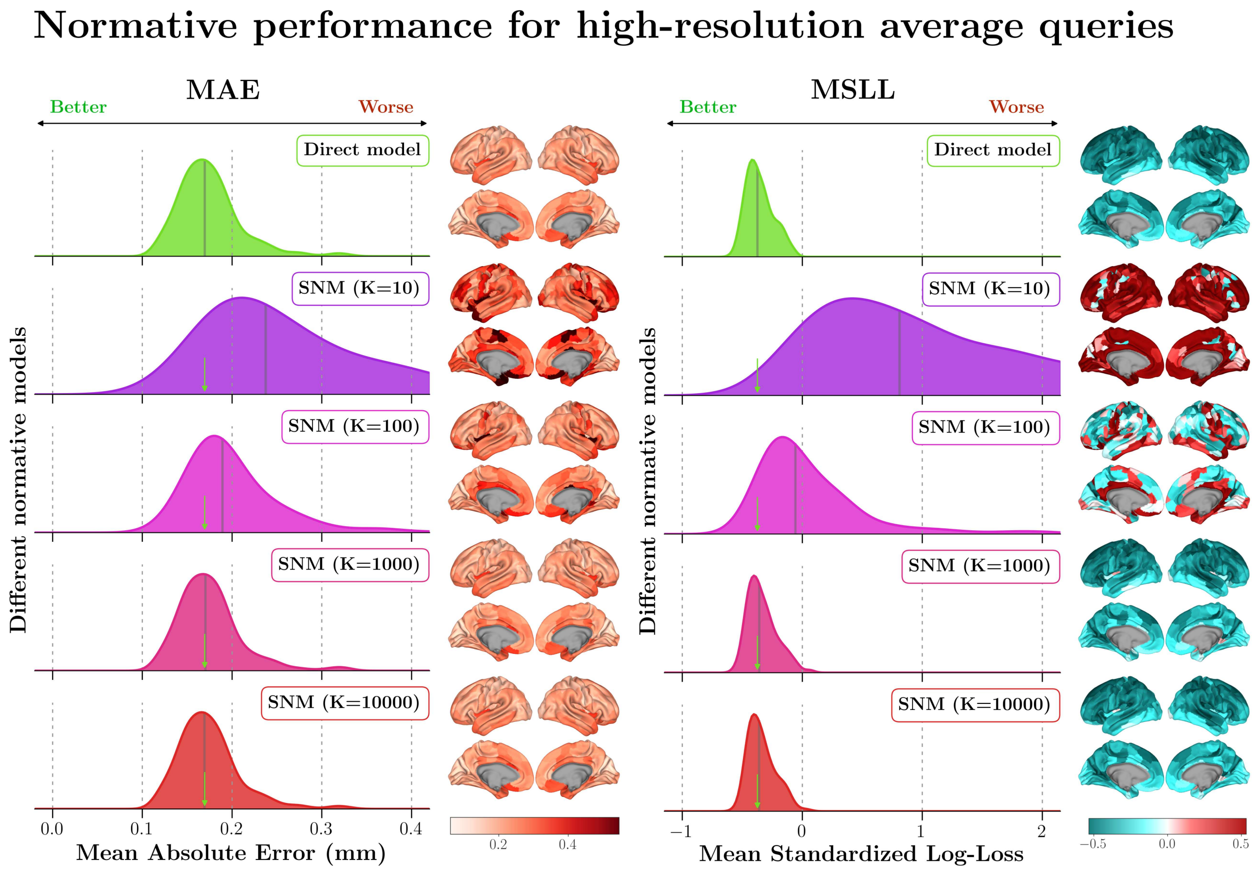

Supplementary Figure. S.4. Comparison of performance for different normative models assessing high-resolution spatial queries. Normative performance is evaluated through out-of-sample assessments of both direct and spectral normative models. The top row displays the performance of the direct model, while the subsequent four rows show the performance of spectral models with 10, 100, 1,000, and 10,000 eigenmodes, respectively. Mean absolute error (MAE, left) and mean standardized log-loss (MSLL, right) quantify model performance. Violin plots illustrate the distribution of performance measures across 400 different spatial queries, each representing an 8mm FWHM Gaussian kernel centered at a high-resolution cortical vertex (one vertex was randomly selected from each region of the Yan400 atlas). Median values are indicated by gray lines on the violin plots. A green arrow highlights the median performance of the direct model in comparison to SNMs. Cortical surface projections show normative performance values for each vertex, depicted within its associated Yan400 region, with shared colorbars across all five rows to facilitate visual comparison.

A.4 Sensitivity Analyses

Several sensitivity analyses were conducted to evaluate the robustness of our findings to various parameters. These analyses can be categorized into two main groups: those assessing the robustness of results against the granularity of the spatial queries, and those evaluating the impact of asymmetry in the spatial queries. These analyses will be respectively detailed in the ensuing sections.

A.4.1 Effect of Spatial Granularity

To evaluate the sensitivity of our findings to the spatial granularity of normative queries, we repeat the evaluations for both regional and high-resolution queries with altered parameters. For regional queries, we assess the impact of increased spatial granularity by using the Yan400 parcellation template, which contains twice as many brain regions as the Yan200 atlas, thereby doubling the spatial granularity of the regional signals. Similarly, the spatial granularity of the high-resolution signals are altered by adjusting the spatial smoothing kernel strength. Specifically, high-resolution evaluations are repeated using a 4mm FWHM Gaussian smoothing kernel, which effectively doubles the spatial granularity of the high-resolution signals.

Supplementary Figure S.5 provides a comparative view of reconstruction accuracy performance as affected by signal granularity. As anticipated, increasing spatial granularity introduces higher spatial frequencies that are captured by higher graph frequencies. This is evident from the slight shift in proportional energy plots, which indicates an increased contribution of higher frequency modes to

medRxiv preprint doi: https://doi.org/10.1101/2025.01.16.25320639; this version posted January 21, 2025. The copyright holder for this preprint (which was not certified by peer review) is the author/funder, who has granted medRxiv a license to display the preprint in perpetuity.
It is made available under a CC-BY 4.0 International license .

signal approximation. Additionally, the results show that the signal reconstruction accuracy for both regional and high-resolution spatial queries reaches a plateau after including the first 1,000 and 2,000 eigenmodes, respectively. Finally, the relatively higher values of SMSE for high-resolution signals with a 4mm FWHM smoothing kernel suggest that high-resolution spectral evaluations are better suited for signals smoothed to 8mm FWHM.

Supplementary Figure S.6 illustrates the impact of regional signal granularity on the goodness of fit of normative models. Repeating the evaluations shown in Figure S.3, we demonstrate that, irrespective of the regional signal granularity, the inclusion of 1,000 eigenmodes in the spectral model achieves performance comparable to that of a direct model. Similarly, Supplementary Figure S.7 repeats the evaluations presented in Figure S.4 and shows that a spectral model with 1,000 modes yields normative estimates comparable to those produced by the direct model. While these results indicate the robustness of the spectral normative framework to changes in spatial granularity, any query used with the spectral model should nevertheless be tested to assess adherence to the low-pass spectral regime (e.g., by evaluating reconstruction accuracy as quantified by metrics such as SMSE).

medRxiv preprint doi: https://doi.org/10.1101/2025.01.16.25320639; this version posted January 21, 2025. The copyright holder for this preprint (which was not certified by peer review) is the author/funder, who has granted medRxiv a license to display the preprint in perpetuity.
It is made available under a CC-BY 4.0 International license .

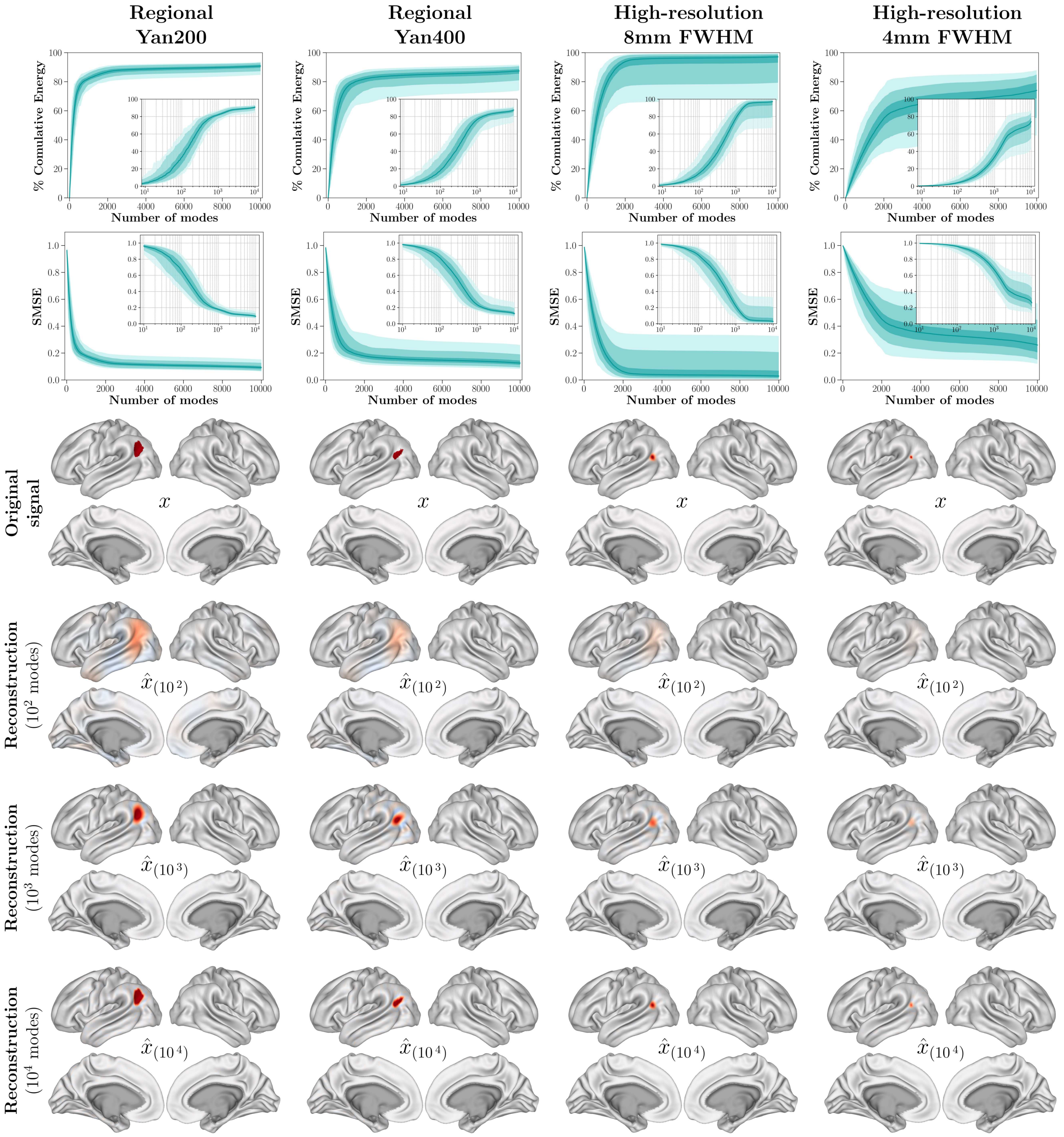

Supplementary Figure. S.5. Signal reconstruction accuracies across different granularity scales. The evaluations presented in Figure 2 are repeated to examine the effect of doubling the spatial granularity of the brain signals. The columns (from left to right) display spectral reconstruction accuracies for regional signals using the Yan200 and Yan400 atlases, as well as for high-resolution signals defined by 8mm FWHM and 4mm FWHM smoothing kernels. In the shaded line plots, the lines represent the median across all observations, while the shades indicate the [25, 75], [5, 95], and [1, 99] percentiles. The first row shows the proportional energy independently contributed by each eigenmode (logarithmic x-axis). The second row presents the standardized mean square error (SMSE) as a function of the number of low-frequency eigenmodes used for reconstruction (logarithmic x-axis for the insets). The third row illustrates one exemplary brain signal from each category, while the last three rows show the same signal reconstructed using 100, 1,000, and 10,000 eigenmodes, respectively.

medRxiv preprint doi: https://doi.org/10.1101/2025.01.16.25320639; this version posted January 21, 2025. The copyright holder for this preprint (which was not certified by peer review) is the author/funder, who has granted medRxiv a license to display the preprint in perpetuity.
It is made available under a CC-BY 4.0 International license .

Normative performance for regional queries: granularity

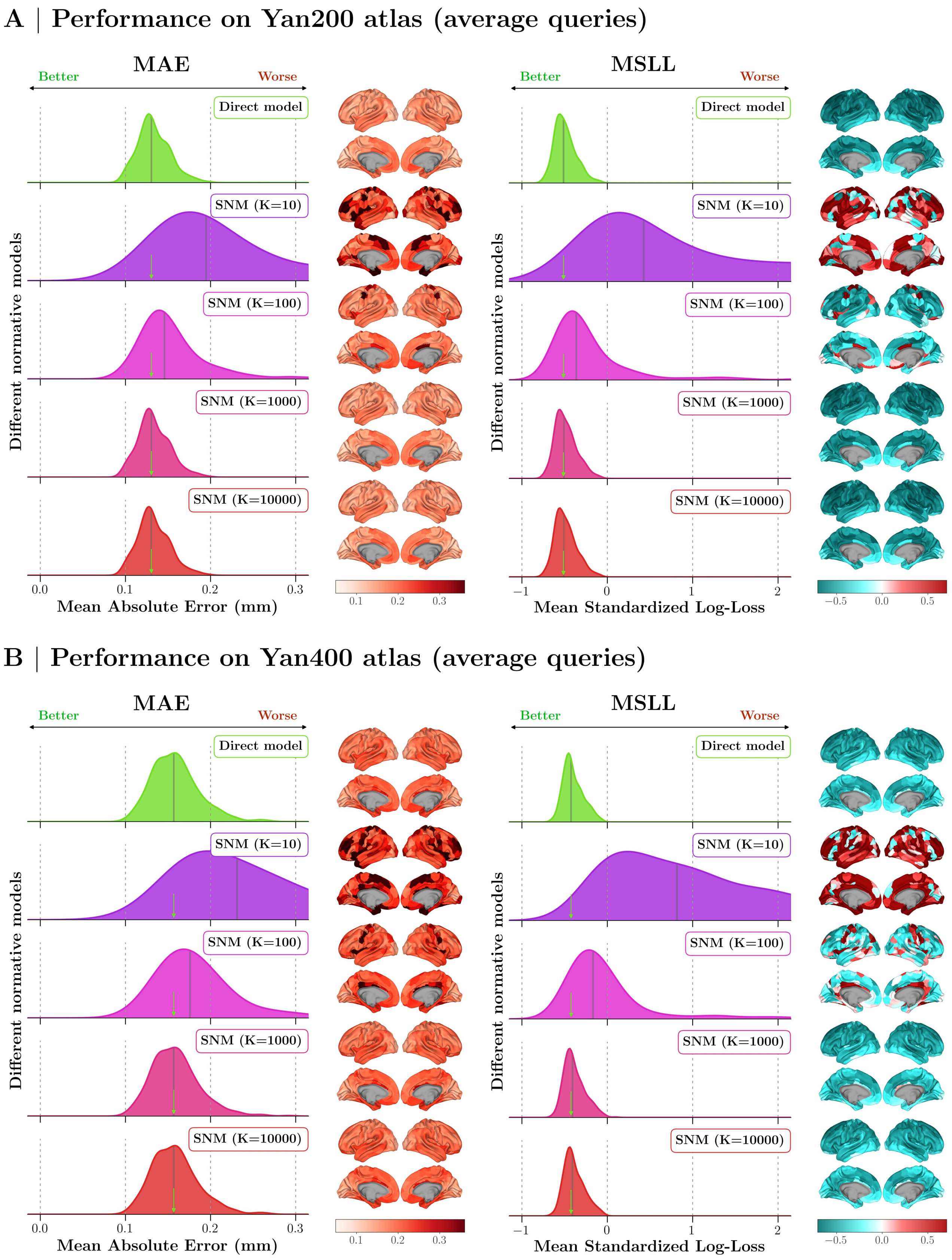

Supplementary Figure. S.6. Sensitivity of normative models assessing regional spatial queries at different granularity scales. Normative performance is evaluated using out-of-sample assessments of both direct and spectral normative models. **(A)** same as Figure S.3, repeated to aid visual comparison. **(B)** Same evaluations as panel A, but on regional queries from Yan400 atlas (increased spatial granularity).

medRxiv preprint doi: https://doi.org/10.1101/2025.01.16.25320639; this version posted January 21, 2025. The copyright holder for this preprint (which was not certified by peer review) is the author/funder, who has granted medRxiv a license to display the preprint in perpetuity.
It is made available under a CC-BY 4.0 International license .

Normative performance for high-resolution queries: granularity

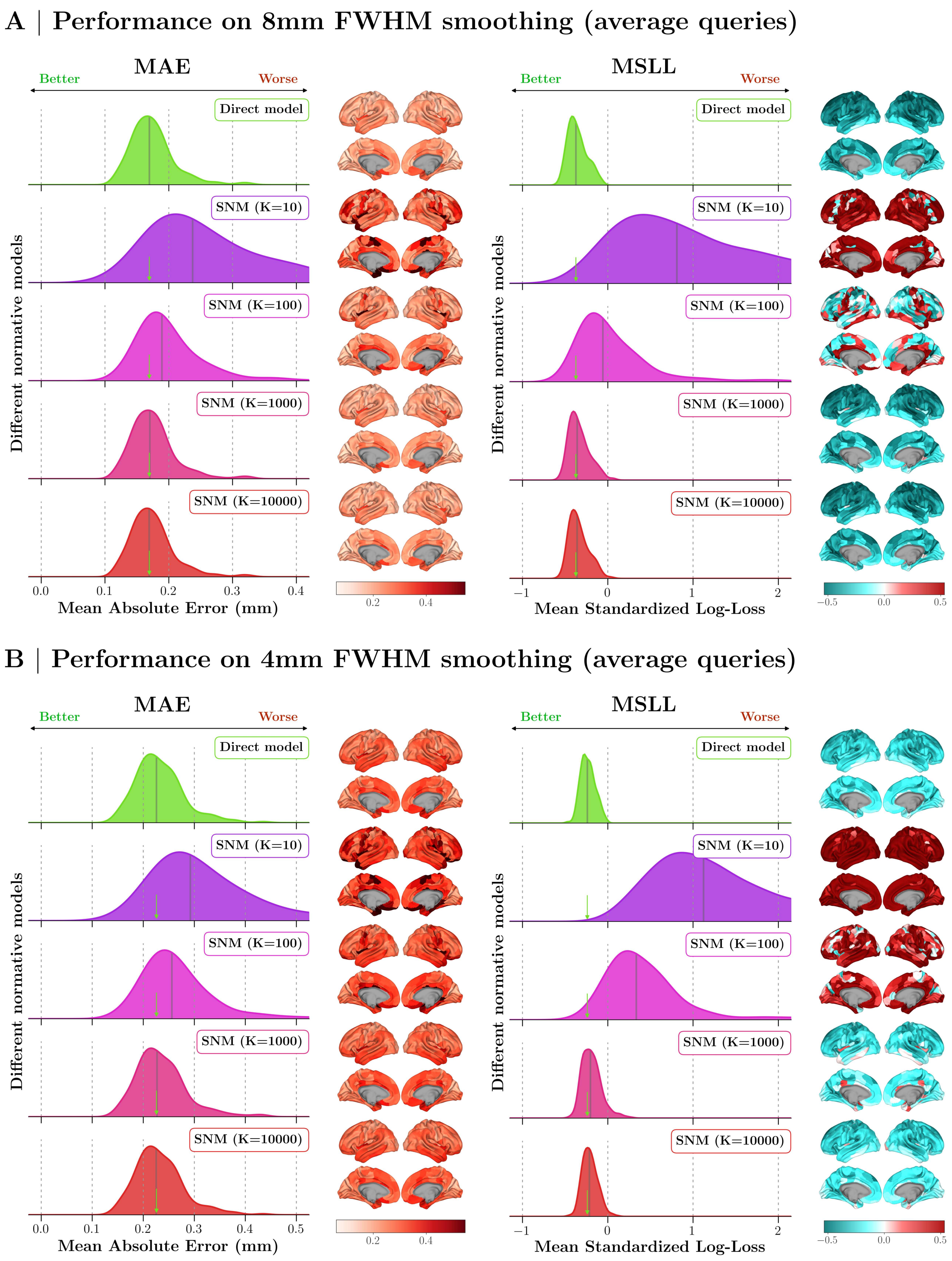

Supplementary Figure. S.7. Sensitivity of normative models assessing high-resolution spatial queries at different granularity scales. Normative performance is evaluated using out-of-sample assessments of both direct and spectral normative models. **(A)** same as Figure S.4, repeated to aid visual comparison. High-resolution spatial queries are generated using an 8mm FWHM Gaussian smoothing kernel. **(B)** Same evaluations as panel A, but on high-resolution queries generated using a 4mm FWHM Gaussian smoothing kernel (increased spatial granularity).

medRxiv preprint doi: https://doi.org/10.1101/2025.01.16.25320639; this version posted January 21, 2025. The copyright holder for this preprint (which was not certified by peer review) is the author/funder, who has granted medRxiv a license to display the preprint in perpetuity.
It is made available under a CC-BY 4.0 International license .

A.4.2 Effect of Spatial Asymmetry

A notable improvement of the spectral normative framework is its spatial versatility. This enables spectral models to extend beyond conventional average-based normative queries and gives them the ability to model a wider range of possible queries. As long as the query of interest adheres to the low-pass spectral regime, it can include various spatial patterns of interest. Asymmetric spatial queries are an interesting example of such patterns. Specifically, spectral normative models can be used to infer abnormal deviations in thickness lateralization by constructing a query that assesses the difference between the average thickness of a region/locus on the left hemisphere and its contralateral counterpart on the right hemisphere. This added benefit requires no extra training time and works out of the box. Hence, we evaluate the sensitivity of our main findings to the spatial asymmetry of normative queries.

We repeat the evaluations for brain-wide, regional, and high-resolution queries after introducing lateralization asymmetry. For brain-wide queries, we use a query that computes the average thickness of the query on the left cortex and subtracts it from the average of the part of the query that lies on the right cortex. For regional queries, given that the Yan atlas provides a homotopic parcellation of the cerebral cortex (i.e., parcels on the left and right cortices are paired), we evaluate 100 spatial normative queries, each assessing the difference in the average thickness of one homotopic parcel pair from Yan200 parcellation. Finally, for high-resolution queries, given that the fs-LR template space is aligned across the left and right cortices, we select pairs of homotopic vertices and construct spatial queries that compare their average thicknesses as described by 8mm FWHM smoothing kernels centered on respective vertex pairs.

Supplementary Figure S.8 provides a comparative view of reconstruction accuracy performance as affected by signal asymmetry. Results indicate that signal asymmetry has negligible impact on eigenmode energy proportions and reconstruction accuracy (quantified by SMSE). Comparing asymmetric queries to average queries (presented in Figure 2), we observe that asymmetric signals require a comparable number of modes for accurate reconstruction as average signals within each spatial query family (brain-wide, regional, and high-resolution). Regardless of signal symmetry, SMSE for the reconstruction of brain-wide, regional, and high-resolution signals respectively reaches below 0.2 after the inclusion of the first 400, 600, and 1,000 eigenmodes. Thus, the reported findings on the number of eigenmodes required to reconstruct brain signals remain consistent regardless of signal asymmetry.

medRxiv preprint doi: https://doi.org/10.1101/2025.01.16.25320639; this version posted January 21, 2025. The copyright holder for this preprint (which was not certified by peer review) is the author/funder, who has granted medRxiv a license to display the preprint in perpetuity.
It is made available under a CC-BY 4.0 International license .

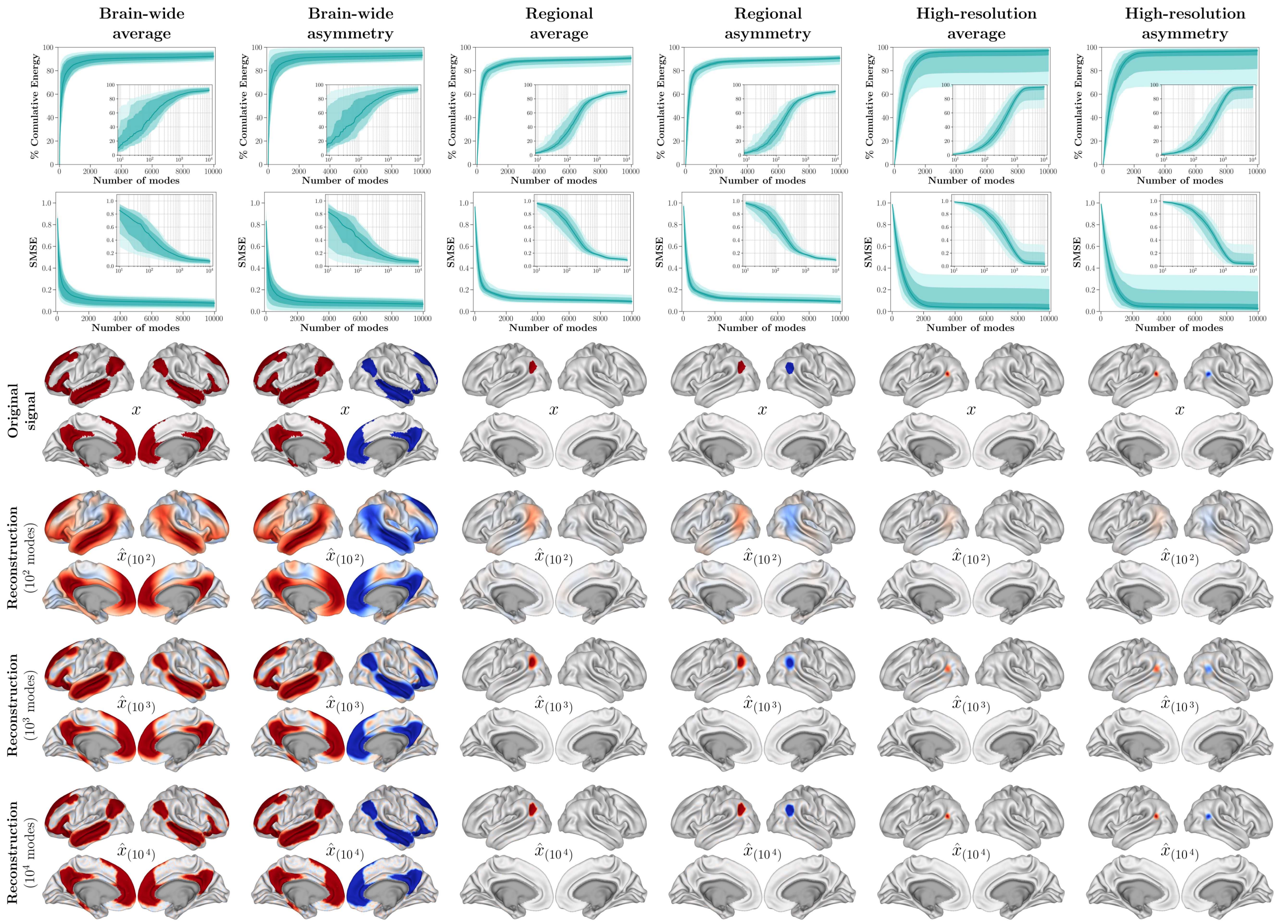

Supplementary Figure. S.8. Signal reconstruction accuracies for average vs. asymmetric signals. The evaluations presented in Figure 2 are repeated to examine the effect of introducing asymmetry to the brain signals. The columns display spectral reconstruction accuracies for pairs of (average, vs. lateralized/asymmetric) brain-wide, regional, and high-resolution signals. In the shaded line plots, the lines represent the median across all observations, while the shades indicate the [25, 75], [5, 95], and [1, 99] percentiles. The first row shows the proportional energy independently contributed by each eigenmode (logarithmic x-axis). The second row presents the standardized mean square error (SMSE) as a function of the number of low-frequency eigenmodes used for reconstruction (logarithmic x-axis for the insets). The third row illustrates one exemplary brain signal from each category, while the last three rows show the same signal reconstructed using 100, 1,000, and 10,000 eigenmodes, respectively.

Supplementary Figures S.9, S.10, and S.11 illustrate the impact of signal asymmetry on the goodness of fit of the associated normative models. Repeating the evaluations shown in Figures S.2, S.3, and S.4 shows that irrespective of the normative modeling framework (direct vs. spectral), goodness of fit, particularly when assessed by MSLL, is lower for lateralized norms. This effect is more pronounced at higher resolutions, suggesting that healthy norms of thickness lateralization are better studied at lower spatial specificity (functional networks or parcels). As the goodness of fit in predicting the central tendency (quantified via MAE) is less affected, we speculate that this reduction in the accuracy of lateralized queries is due to the misaligned gyrification patterns between contralaterally aligned high-resolution vertices, rendering lateralization evaluations less meaningful at higher resolutions.

Despite this effect, which was not specific to the spectral model, we observe that, similar to the main findings, the inclusion of 100 eigenmodes is sufficient to model brain-wide normative thickness ranges with the spectral model, achieving performance on par with the direct model. As with the main findings, the number of modes required to achieve comparable performance to that of a direct model at the resolution of regional or vertex-wise queries increases to 1,000 eigenmodes. These sensitivity evaluations demonstrate that our findings are robustly replicable for signals with asymmetry, and that the inclusion of 1,000 eigenmodes can provide comparable performance to that of a conventional model across a wide range of spatial queries.

medRxiv preprint doi: https://doi.org/10.1101/2025.01.16.25320639; this version posted January 21, 2025. The copyright holder for this preprint (which was not certified by peer review) is the author/funder, who has granted medRxiv a license to display the preprint in perpetuity.
It is made available under a CC-BY 4.0 International license .

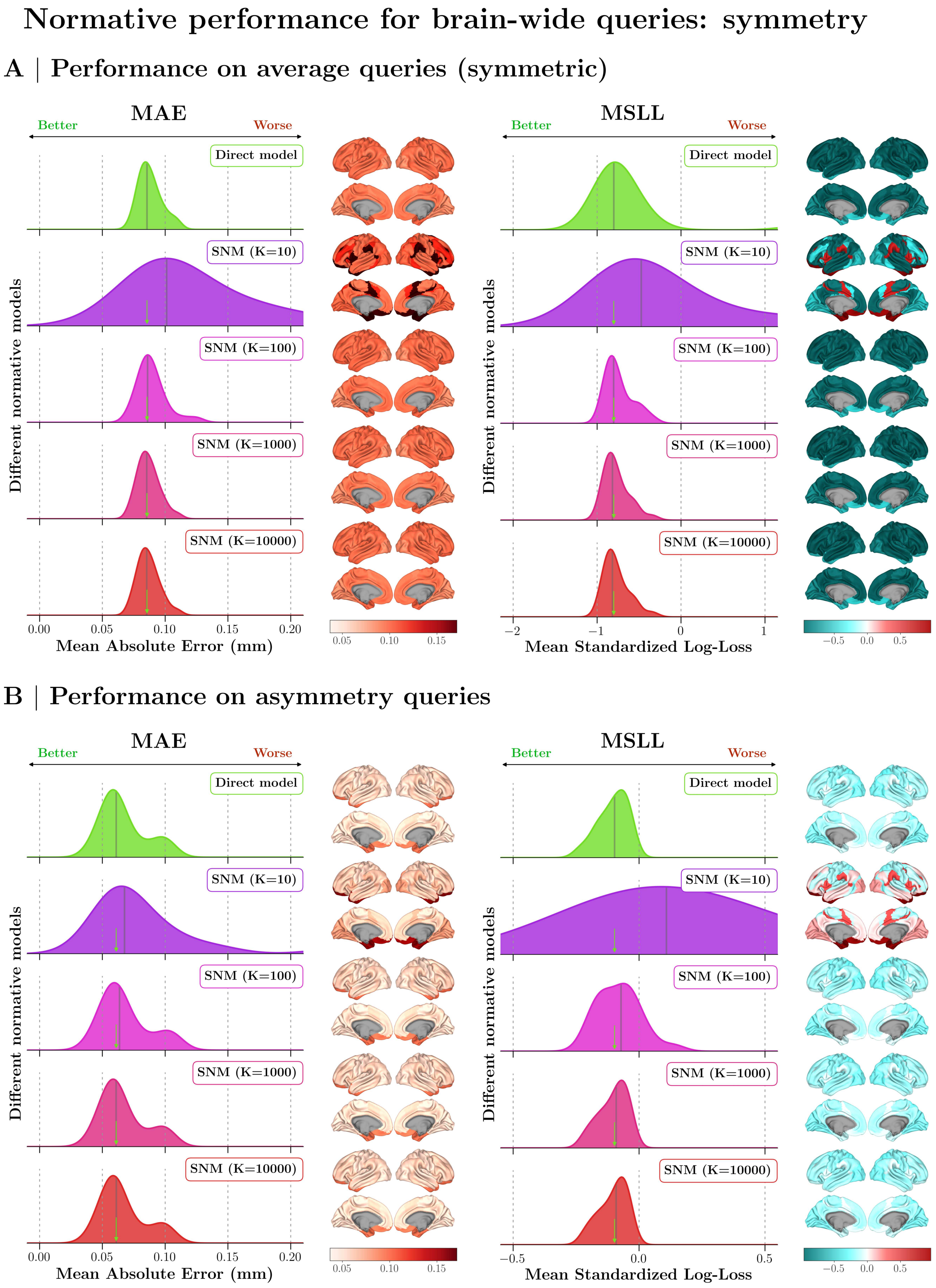

Supplementary Figure. S.9. Sensitivity of normative models to assessing lateralized brain-wide spatial queries. Normative performance is evaluated using out-of-sample assessments of both direct and spectral normative models. **(A)** same as Figure S.2, repeated to aid visual comparison. **(B)** Same evaluations as panel A, but on lateralized counterparts of the brain-wide queries.

medRxiv preprint doi: https://doi.org/10.1101/2025.01.16.25320639; this version posted January 21, 2025. The copyright holder for this preprint (which was not certified by peer review) is the author/funder, who has granted medRxiv a license to display the preprint in perpetuity.
It is made available under a CC-BY 4.0 International license .

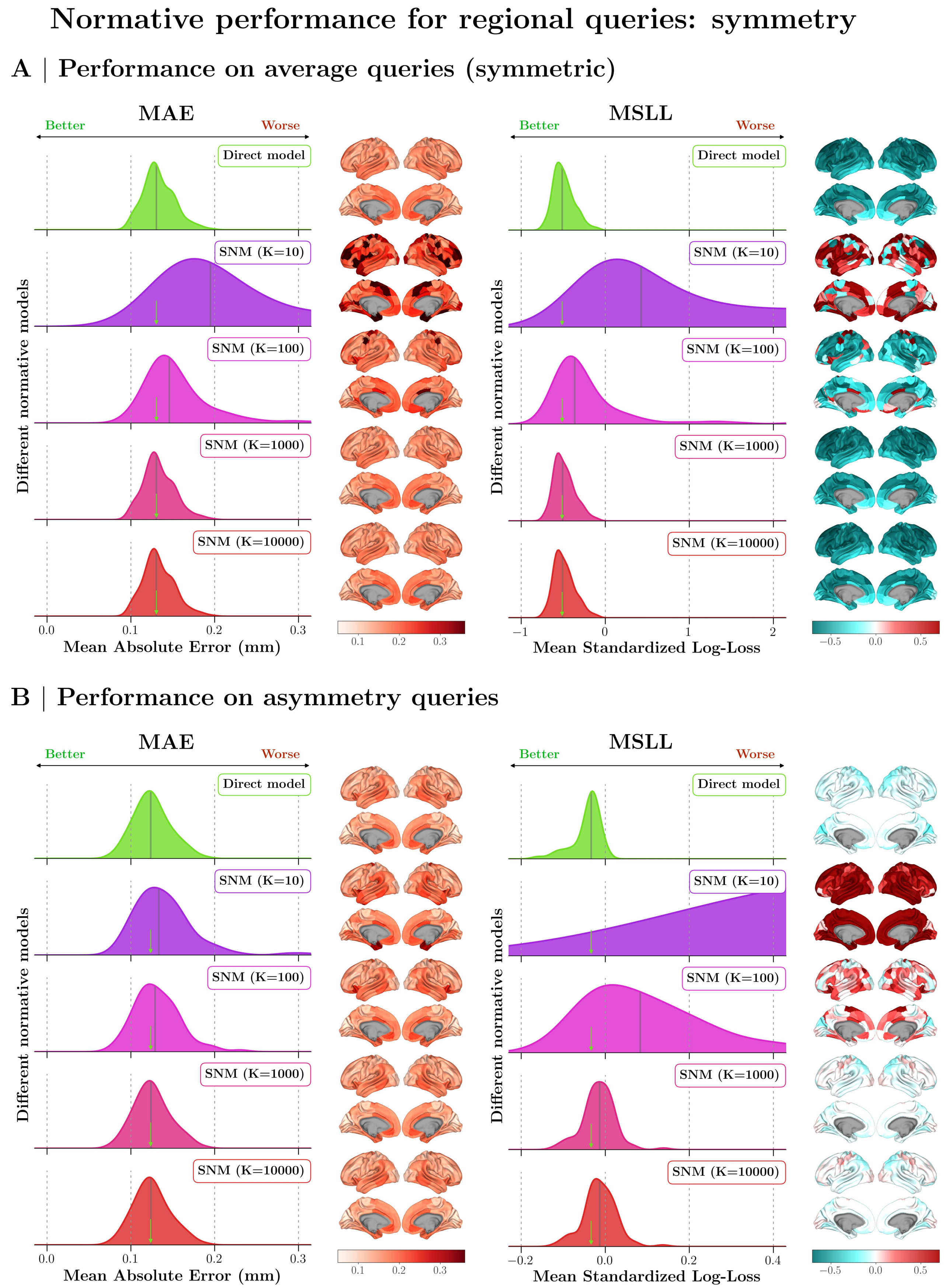

Supplementary Figure. S.10. Sensitivity of normative models to assessing lateralized regional spatial queries. Normative performance is evaluated using out-of-sample assessments of both direct and spectral normative models. **(A)** same as Figure S.3, repeated to aid visual comparison. **(B)** Same evaluations as panel A, but on lateralized counterparts of the regional queries.

medRxiv preprint doi: https://doi.org/10.1101/2025.01.16.25320639; this version posted January 21, 2025. The copyright holder for this preprint (which was not certified by peer review) is the author/funder, who has granted medRxiv a license to display the preprint in perpetuity.
It is made available under a CC-BY 4.0 International license .

Normative performance for high-resolution queries: symmetry

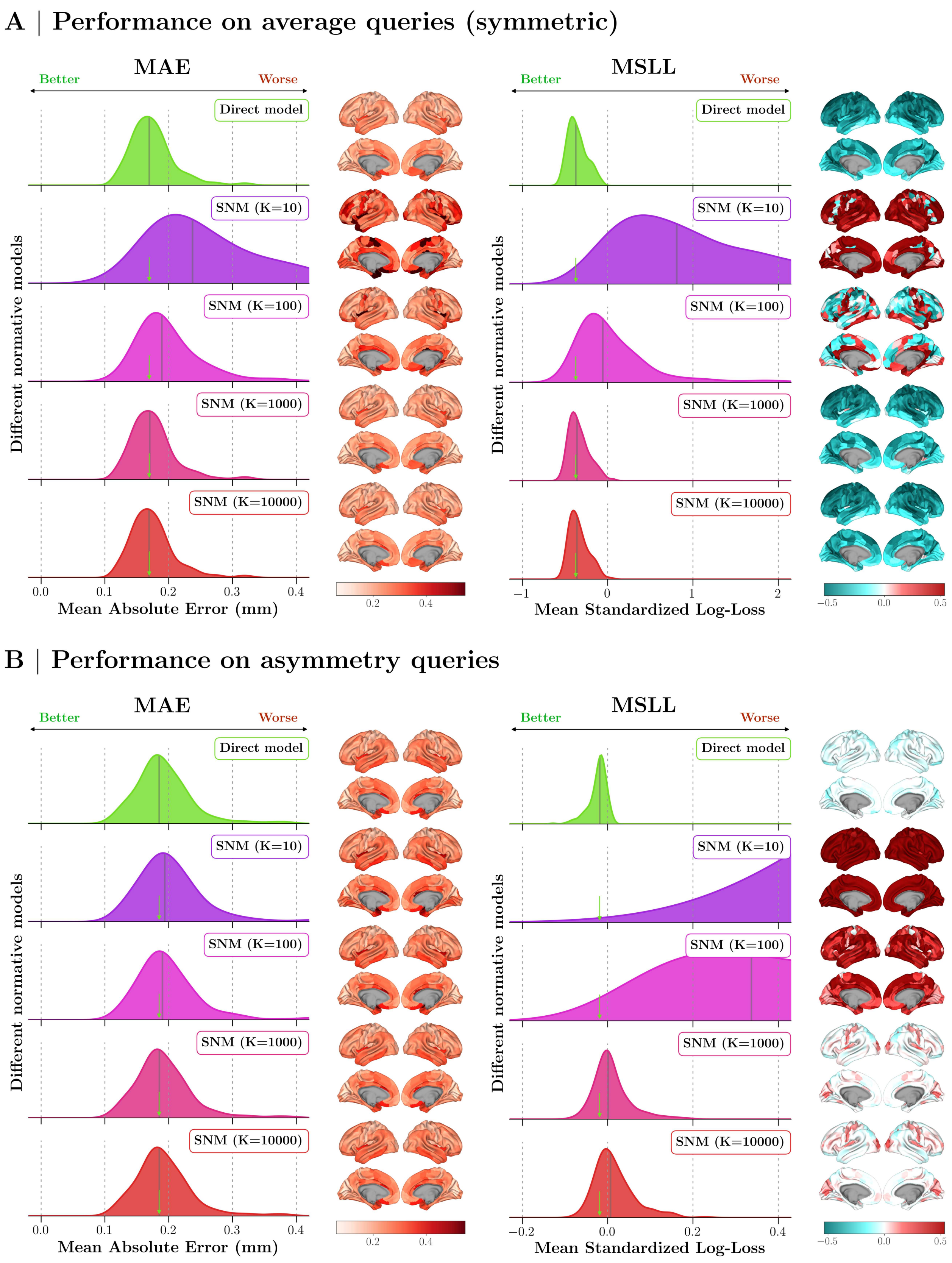

Supplementary Figure. S.11. Sensitivity of normative models to assessing lateralized high-resolution spatial queries. Normative performance is evaluated using out-of-sample assessments of both direct and spectral normative models. **(A)** same as Figure S.4, repeated to aid visual comparison. **(B)** Same evaluations as panel A, but on lateralized counterparts of the high-resolution queries.

medRxiv preprint doi: https://doi.org/10.1101/2025.01.16.25320639; this version posted January 21, 2025. The copyright holder for this preprint (which was not certified by peer review) is the author/funder, who has granted medRxiv a license to display the preprint in perpetuity.
It is made available under a CC-BY 4.0 International license .

A.5 Reconstruction Residuals

In Figure 2, cortical projections of exemplary brain signals were presented alongside the original signal (last four rows). Supplementary Figure S.12 shows the residuals of low-pass filtered graph approximations of the same signals. For four different low-pass approximations ($k = 10, 10^2, 10^3, 10^4$), the approximation residuals ($x - \hat{x}_{(k)}$) are projected onto the cortical surface. As anticipated, the magnitude of the approximation error (indicated by color intensity in the cortical projections of residuals) decreases as the number of incorporated eigenmodes increases. Furthermore, with the inclusion of more modes, only regions containing high spatial frequency information (such as the transition loci along parcellation borders) have higher approximation errors. This verifies our expectations that low-pass filtering by eigenmodes can accurately approximate smooth signals (i.e. lower graph frequencies).

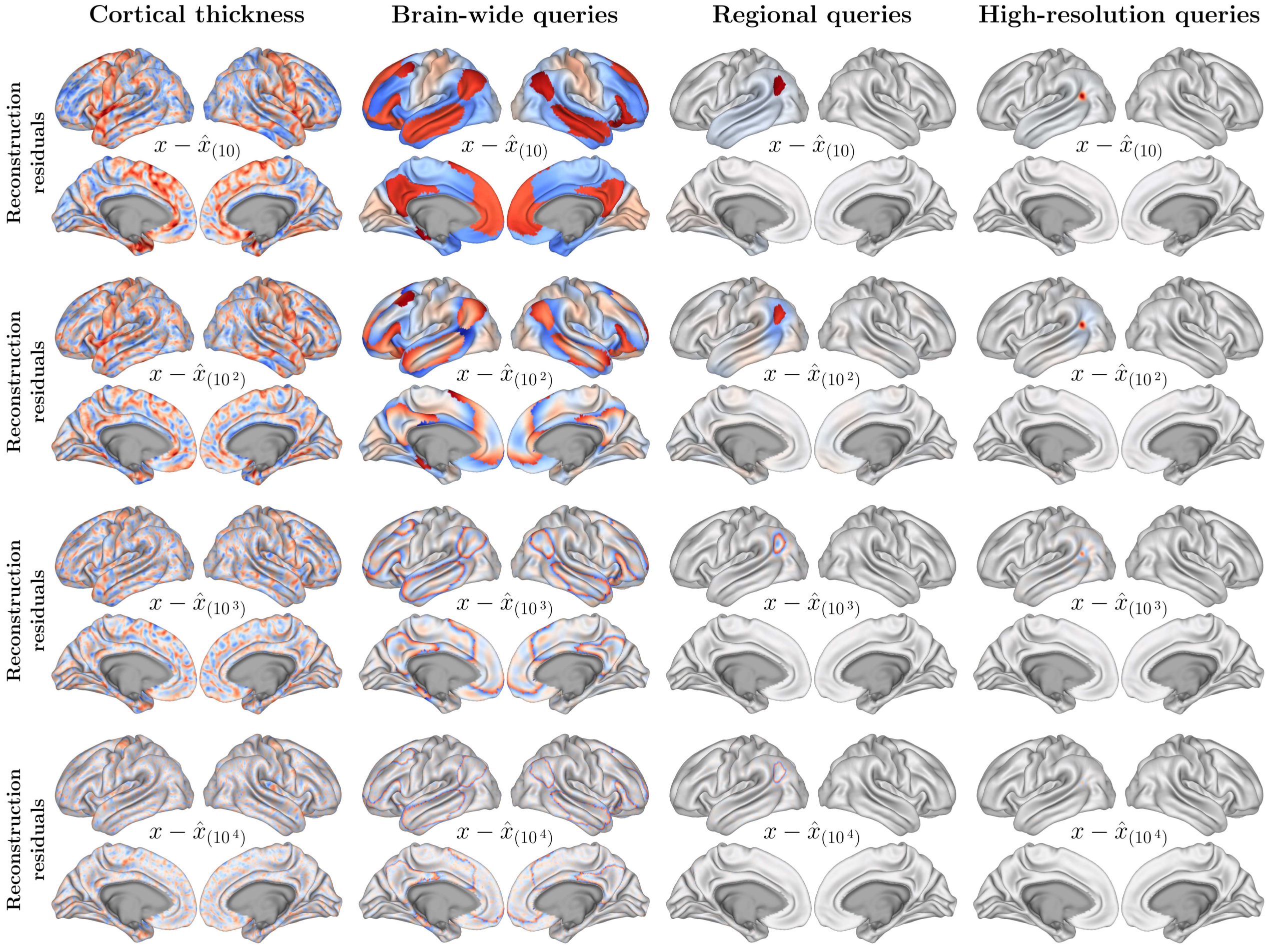

Supplementary Figure. S.12. Signal reconstruction residuals. The columns (from left to right) display the cortical projections of approximation residuals for individual cortical thickness signals, as well as brain-wide, regional, and high-resolution spatial queries (similar to Figure 2). The rows show the residuals for the same signal reconstructed using 10, 100, 1,000, and 10,000 eigenmodes, respectively.

medRxiv preprint doi: https://doi.org/10.1101/2025.01.16.25320639; this version posted January 21, 2025. The copyright holder for this preprint (which was not certified by peer review) is the author/funder, who has granted medRxiv a license to display the preprint in perpetuity.
It is made available under a CC-BY 4.0 International license .

A.6 Within-group Cognitive Associations

To assess the significance of cognitive associations within each cohort, we repeated the analyses from Section 2.5 SNM Uncovers Cortical Signatures of Atrophy in Alzheimer's Disease on the HC, MCI, and AD subsets. For healthy controls (HC), high-resolution cortical associations did not reach significance in any region after FDR correction (Supplementary Figure S.13), and ETVC showed no association with cognitive performance, irrespective of the z-threshold. In the MCI cohort, ETVC was similarly unrelated to cognitive impairment (Supplementary Figure S.14), but regional analyses identified significant atrophy in the left entorhinal cortex and left frontal pole to be associated with cognitive impairment. In contrast, the AD cohort displayed widespread neocortical atrophy patterns significantly linked to cognitive impairment (Supplementary Figure S.15), with reductions in the temporal pole, superior and middle temporal gyri, entorhinal cortex, parahippocampal cortex, precuneus, supramarginal gyrus, and superior, middle, and inferior frontal gyri. Additionally, ETVC showed a strong association with cognitive impairment in the AD cohort, specific to extremely thin vertices. Interestingly, this predictive capacity diminished when the z-score threshold was raised to zero or any positive value, with optimal predictions achieved between z-score thresholds of -2 and -3. Together, these findings suggest that the derived normative atrophy marker of cognitive impairment is specific to AD and most sensitive to extreme signs of atrophy. However, it should be noted that limited variability in cognitive performance within HC and MCI cohorts has potentially reduced the sensitivity for detecting smaller effects in these groups.

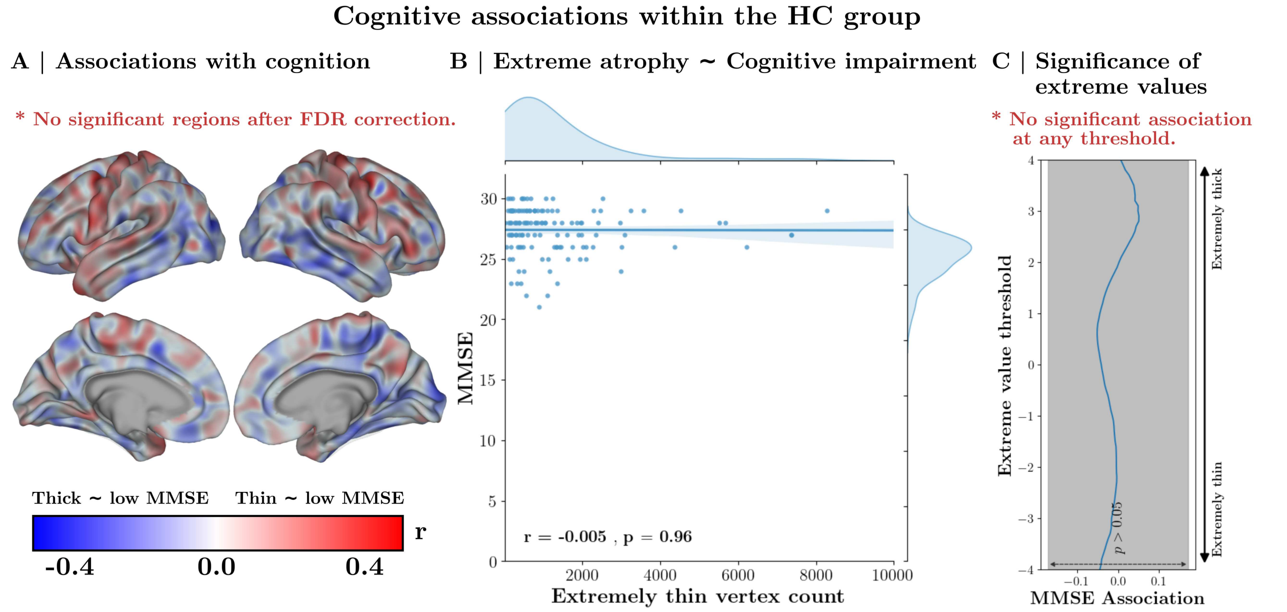

Supplementary Figure. S.13. Cognitive Association Tests within the Healthy Cohort (HC). (A) Vertex-level normative assessments were tested for linear associations with cognitive performance (MMSE), but no vertices reached significance after nonparametric FDR correction. **(B)** Extreme value statistics for severe atrophy (ETVC, z-score threshold of -1.96) were assessed for their predictive capability on cognitive performance, revealing no sensitivity of ETVC to cognitive variations in HC. **(C)** A range of alternative z-score thresholds was tested, demonstrating that ETVC does not predict cognitive performance in HC, regardless of threshold choice.

medRxiv preprint doi: https://doi.org/10.1101/2025.01.16.25320639; this version posted January 21, 2025. The copyright holder for this preprint (which was not certified by peer review) is the author/funder, who has granted medRxiv a license to display the preprint in perpetuity.
It is made available under a CC-BY 4.0 International license .

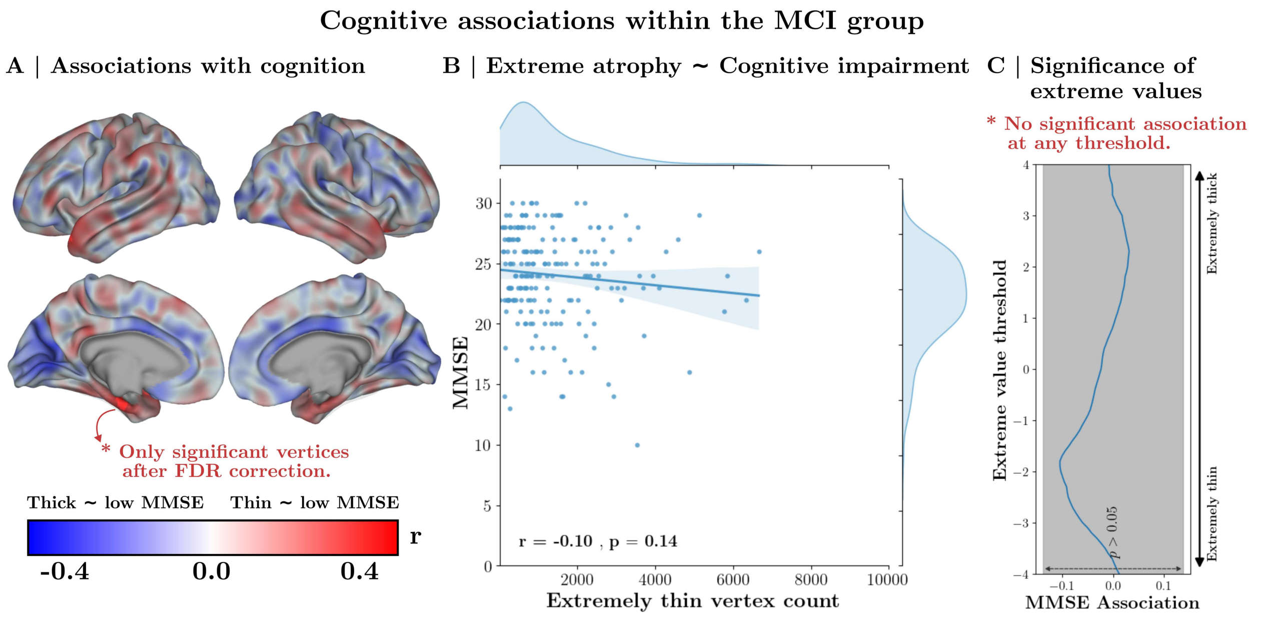

Supplementary Figure. S.14. Cognitive Association Tests within the MCI Cohort. (A) Vertex-level normative assessments were tested for linear associations with cognitive performance (MMSE) within the MCI cohort. A limited set of vertices within the entorhinal cortex reached statistical significance, indicating that extreme atrophy in this region may serve as a marker of cognitive impairment. **(B)** Extreme value statistics for severe atrophy (ETVC, z-score threshold of -1.96) were evaluated for their predictive capability on cognitive performance, showing no sensitivity of ETVC to cognitive variations within MCI. **(C)** Testing a range of alternative z-score thresholds confirmed that ETVC does not predict cognitive performance in MCI, regardless of the chosen threshold.

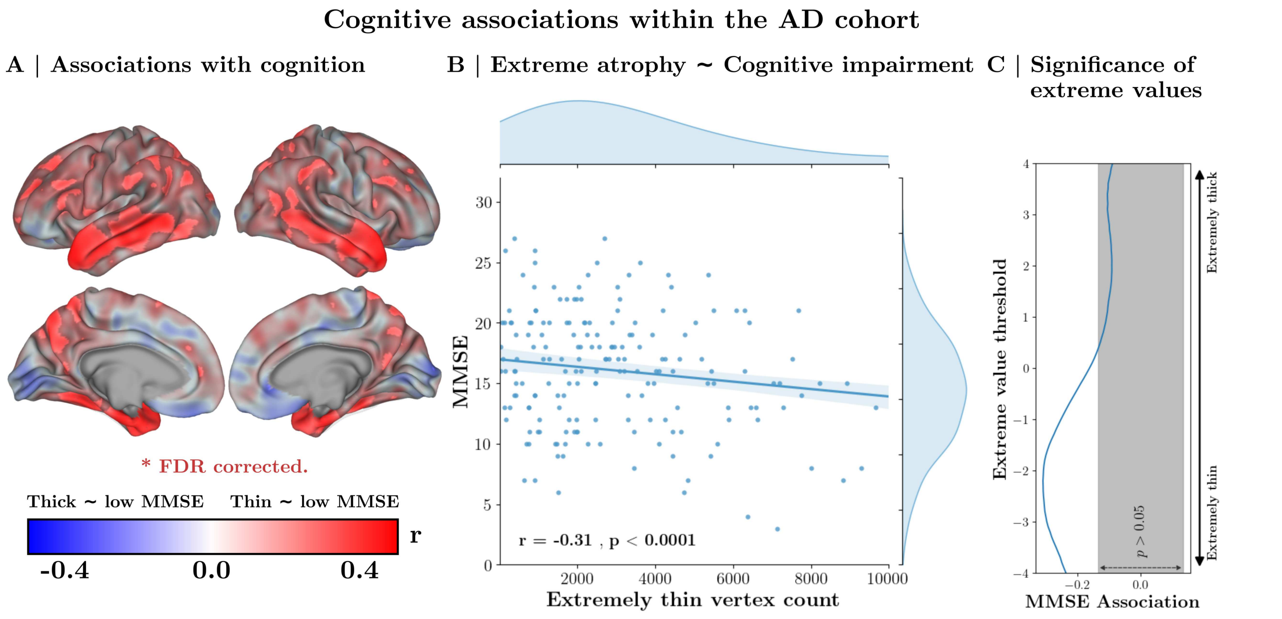

Supplementary Figure. S.15. Cognitive Association Tests within the AD Cohort. (A) Vertex-level normative assessments were tested for linear associations with cognitive performance (MMSE) within the AD cohort. A widespread set of cortical regions reached statistical significance, highlighting extensive brain-wide associations as markers of AD-related cognitive impairment. **(B)** Extreme value statistics for severe atrophy (ETVC, z-score threshold of -1.96) were evaluated for predictive capability regarding cognitive performance, revealing significant predictive power of ETVC to capture cognitive variations within the AD cohort. **(C)** Testing across a range of alternative z-score thresholds indicated that only ETVCs with a negative z-threshold significantly predicted cognitive performance in the AD cohort. Notably, extreme thinning (z-thresholds from -2 to -3) proved to be the strongest predictor of cognitive impairment.

medRxiv preprint doi: https://doi.org/10.1101/2025.01.16.25320639; this version posted January 21, 2025. The copyright holder for this preprint (which was not certified by peer review) is the author/funder, who has granted medRxiv a license to display the preprint in perpetuity.
It is made available under a CC-BY 4.0 International license .

A.7 Brain-wide Cognitive Associations

To highlight the benefits of inferring high-resolution spatial norms, we compare the predictive performance of the ETVC normative measure derived from high-resolution brain charting with that of a brain-wide normative z-score computed from a spatially coarse brain chart of mean cortical thickness (averaged across the entire cortex). Using the fine-tuned SNM, we calculate individual z-scores for deviations in average cortical thickness and replicate the evaluations from Figures 5C, S.13B, S.14B, and S.15B. As shown in Figure S.16, the results consistently indicate that while brain-wide normative measures can predict cognitive impairments, high-resolution ETVC measures outperform them. Specifically, ETVC achieves higher prediction accuracy in the entire sample (ETVC: $|r| = 0.45$, brain-wide: $|r| = 0.31$) and in the AD subsample (ETVC: $|r| = 0.31$, brain-wide: $|r| = 0.23$). Notably, both normative metrics were specifically sensitive to AD-related cognitive differences as neither predicted cognition in the HC or MCI subsets.

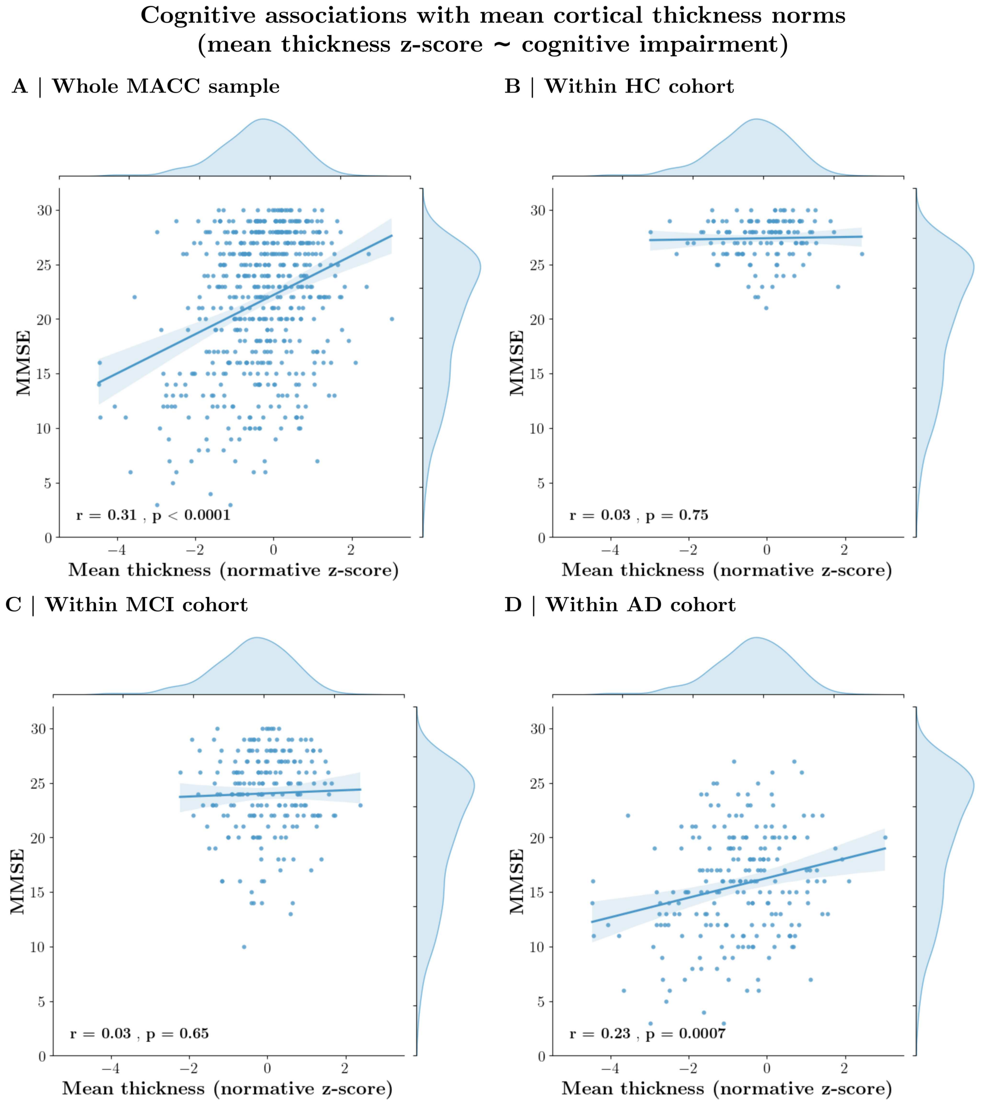

Supplementary Figure. S.16. Cognitive Association Tests with a Coarse Brain-Wide Normative Metric. Normative deviations (z-scores) in mean cortical thickness were evaluated for their predictive capability regarding cognitive performance (quantified by MMSE). Predictive performance was assessed in **(A)** the entire MACC dataset, as well as within the **(B)** HC, **(C)** MCI, and **(D)** AD cohorts individually.

A.8 Heterogeneity Landscape Subgroups

Figure 6D presents snapshots of cortical deviation patterns across various regions within the 2-dimensional atrophy landscape. To more comprehensively illustrate the overall cortical signature in this landscape, we have provided four supplementary videos (one each for the whole sample, HC, MCI, and AD subsets). These videos display changes in the cortical deviation patterns as the local region is gradually shifted over a circular area within the 2-dimensional space.
